# Disentangling the resistant mechanism of Fusarium wilt TR4 interactions with different cultivars and its elicitor application

**DOI:** 10.3389/fpls.2023.1145837

**Published:** 2023-03-02

**Authors:** Guang-Dong Zhou, Ping He, Libo Tian, Shengtao Xu, Baoming Yang, Lina Liu, Yongfen Wang, Tingting Bai, Xundong Li, Shu Li, Si-Jun Zheng

**Affiliations:** ^1^ Yunnan Key Laboratory of Green Prevention and Control of Agricultural Transboundary Pests, Agricultural Environment and Resources Institute, Yunnan Academy of Agricultural Sciences, Kunming, Yunnan, China; ^2^ Center For Potato Research, Resource Plant Research Institute, Yunnan University, Kunming, Yunnan, China; ^3^ State Key Laboratory for Conservation and Utilization of Bio-Resources in Yunnan, Ministry of Education Key Laboratory of Agriculture Biodiversity for Plant Disease Management, College of Plant Protection, Yunnan Agricultural University, Kunming, Yunnan, China; ^4^ Institute of Tropical and Subtropical Industry Crops, Yunnan Academy of Agricultural Sciences, Baoshan, China; ^5^ Bioversity International, Kunming, Yunnan, China

**Keywords:** cultivar, Fusarium wilt of banana (FWB), Tropical Race 4 (TR4), induced resistance, elicitor, starch granule, isotianil

## Abstract

Fusarium wilt of banana, especially Tropical Race 4 (TR4) is a major factor restricting banana production. Developing a resistant cultivar and inducing plant defenses by elicitor application are currently two of the best options to control this disease. Isotianil is a monocarboxylic acid amide that has been used as a fungicide to control rice blast and could potentially induce systemic acquired resistance in plants. To determine the control effect of elicitor isotianil on TR4 in different resistant cultivars, a greenhouse pot experiment was conducted and its results showed that isotianil could significantly alleviate the symptoms of TR4, provide enhanced disease control on the cultivars ‘Baxi’ and ‘Yunjiao No.1’ with control effect 50.14% and 56.14%, respectively. We compared the infection processes in ‘Baxi’ (susceptible cultivars) and ‘Yunjiao No.1’ (resistant cultivars) two cultivars inoculated with pathogen TR4. The results showed that TR4 hyphae could rapidly penetrate the cortex into the root vascular bundle for colonization, and the colonization capacity in ‘Baxi’ was significantly higher than that in ‘Yunjiao No.1’. The accumulation of a large number of starch grains was observed in corms cells, and further analysis showed that the starch content in ‘Yunjiao No. 1’ as resistant cultivar was significantly higher than that in ‘Baxi’ as susceptible cultivar, and isotianil application could significantly increase the starch content in ‘Baxi’. Besides, a mass of tyloses were observed in the roots and corms and these tyloses increased after application with isotianil. Furthermore, the total starch and tyloses contents and the control effect in the corms of ‘Yunjiao No.1’ was higher than that in the ‘Baxi’. Moreover, the expression levels of key genes for plant resistance induction and starch synthesis were analyzed, and the results suggested that these genes were significantly upregulated at different time points after the application of isotianil. These results suggest that there are significant differences between cultivars in response to TR4 invasion and plant reactions with respect to starch accumulation, tyloses formation and the expression of plant resistance induction and starch synthesis related genes. Results also indicate that isotianil application may contribute to disease control by inducing host plant defense against TR4 infection and could be potentially used together with resistant cultivar as integrated approach to manage this destructive disease. Further research under field conditions should be included in the next phases of study.

## 1 Introduction

Bananas, the most traded tropical and subtropical fruit (Li et al., 2019; Zou and Fan, 2022), are also fourth staple crop after wheat, corn and rice (Nayar, 2010), providing food source for approximately 400 million population worldwide (Dusunceli, 2017). However, banana industry is seriously threatened by Fusarium wilt of banana (FWB) which is a soil-borne vascular bundle disease caused by *Fusarium oxysporum* f. sp. *cubense* (Foc) (Ploetz, 2006a; Ploetz, 2015; Dita et al., 2018). On the basis of difference in pathogenicity of *Foc*, it can be divided into 4 physiological races (*Foc* 1, *Foc* 2, *Foc* 3 and *Foc* 4) and *Foc* 4 can be divided into subtropical race 4 (STR4) and tropical race 4 (TR4) (Ploetz, 2006b; Karangwa et al., 2018). In the 1950s’, the FWB caused by Foc1 was successful controlled by replaced the ‘Gros Michel’ (disease-susceptible cultivar) with the ‘Cavendish’(disease-resistant cultivar) (Ploetz, 2006b). In the 1990s, the banana industry was again in crisis with the advent of TR4 (Ploetz, 2006b). In the past decades, TR4 gradually has spread to the surrounding countries such as the Philippines and Malaysia (Hwang and Ko, 2004; Ploetz, 2006b). Then it expanded to countries and regions such as Australia, the Middle East, India and Africa (Thangavelu and Mustaffa, 2010; Butler, 2013; Ploetz et al., 2015). In recent years, it has been found in Jordan (García-Bastidas et al., 2014), Lebanon (Ordoñez et al., 2016), Israel (Maymon et al., 2018), Mozambique (García-Bastidas et al., 2014), Pakistan (Ordoñez et al., 2016), Puerto Rico (Garcia et al., 2018), Miyako Island in Okinawa, Japan (Nitani et al., 2018), India (Thangavelu et al., 2019), Mayotte (Aguayo et al., 2020), Colombia (Bastidas et al., 2020) and Peru (Acuña et al., 2021). As TR4 continues to spread rapidly around the world (Dita et al., 2018; Zheng et al., 2018; Pegg et al., 2019), it is essential to take actions to stop its further spread and to have comprehensive management approaches. Nowadays, although historical experience has shown that disease-resistance breeding is a particularly effective way to control FWB (Li et al., 2015; Bubici1 et al., 2019; Zorrilla-Fontanesi et al., 2020), no completely immune TR4 cultivar has been incorporated into agricultural production, because of the major challenge faced to breed disease-resistant cultivars in traditional ways due to the peculiarities of triploids of banana plants.

In the natural environment, in order to prevent pathogenic infection, plants not only form a physical barrier on the surface, but also have various internal immune responses. Plants are able to induce broad defense reactions by pathogens in their surroundings (Choudhary et al., 2007). So, activating its inherent defense by specific elicitors would be an effective way to protect plants from disease (Ward et al., 1991; Pieterse et al., 1998b). Therefore, most researchers prefer the plant-induced resistance as a new type of plant disease control strategy (Eschen-Lippold et al., 2010; Kurth et al., 2014; Dorneles et al., 2018; Sopeña-Torres et al., 2018), which may also become a new sustainable plant protection approach in the future (Roberts and Taylor, 2016).Today, many bacterial, fungal and chemical inducers that induce plant defenses to control crop disease have been commercialized (Verhagen et al., 2004; Takahashi et al., 2006). However, so far there is no study on exogenous inducers on FWB. Whether these exogenous elicitors can induce bananas to acquire systemic resistance to FWB is still unknown.

According to the molecular mechanism of induction, induced resistance is divided into systemic acquired resistance (SAR) and induced systemic resistance (ISR) (Pieterse et al., 2009). SAR, which depends on salicylic (SA) and its associated systemic immune responses have been confirmed in some plants (Pieterse et al., 2002; Fu and Dong, 2013; Bektas and Eulgem, 2014), such as, enhances the expression of pathogenesis-related (*PR*) genes (Loon et al., 2006). *PR* proteins, with antibacterial activity outside the cell,can directly act on pathogen (Loon et al., 2006). *NPR1*, a key gen regulator for transducing the SA signaling and activating PR gene expression in the pathway (Dong, 2004; Grant and Lamb, 2006), and both exogenous SA application and pathogen infection may lead to enhanced expression of the NPR1 gene of the SAR pathway in plants (Cao et al., 1997; Ryals et al., 1997). In contrast to SAR, ISR relies primarily on jasmonic acid (JA) and ethylene (ET) pathways (Loon et al., 1998; Pieterse et al., 1998a; Pieterse et al., 2002; Choudhary et al., 2007; Pieterse et al., 2012; Pieterse et al., 2014). Although SAR and ISR are significantly different, studies have shown that ISR also requires NPR1 (Pieterse et al., 2014; Nie et al., 2017). ET is synthesized from the amino acid methionine by a pathway requiring *SAMS* (S-adenosylmethionine synthetase), *ACS* [1-aminocyclopropane-1-carboxylic acid (ACC) synthase] and *ACO* (ACC oxidase), and ACS is a key synthetase gen in this pathway (Sauter et al., 2013; Wang et al., 2013; Dubois et al., 2018). In general, when ISR is activated, the expression of genes involved in ET biosynthesis (e.g., *ACS*) and signaling (e.g., *ERF1*, ethylene response factor 1) is usually upregulated (Shoresh et al., 2005; Ribaudo et al., 2006; Poupin et al., 2016). The basic helix-loop-helix (*bHLH*) transcription factor (TF) *MYC2* as a major regulator in the JA pathway, coordinates plant resistance to pathogens through JA-mediated defense responses (Lorenzo et al., 2004; Kazan and Manners, 2013; Du et al., 2017; Liu et al., 2019). In addition, both ET and JA pathways activation synergistically induce plant response to pathogens by ERF1 transcription factor (Zhou et al., 2022).

Starch is a decisive factor for plants to adapt to abiotic stress (Thalmann and Santelia, 2017), and often shows very obvious plasticity when different plant tissues face stresses (Cuellar-Ortiz et al., 2008; Yin et al., 2009; Morais et al., 2019). Bananas plants with high starch content in corm are more resistant to FWB than those with low starch content (Dong et al., 2019). It is well known that ADP-glucose pyrophosphorylase (*AGPase*), Starch branching enzyme (*SBE*) and granule-binding starch synthase (*GBSS*) are key enzymes in the starch biosynthesis pathway, and *AGPase* plays an important role in crop heat tolerance (Saripalli and Gupta, 2015).The activity of *GBSS* within granules is the main determinant of amylose content (Seung, 2020). *SBE* is a key enzyme in pullulan synthesis (Li and Gilbert, 2016).

**Figure 1:**
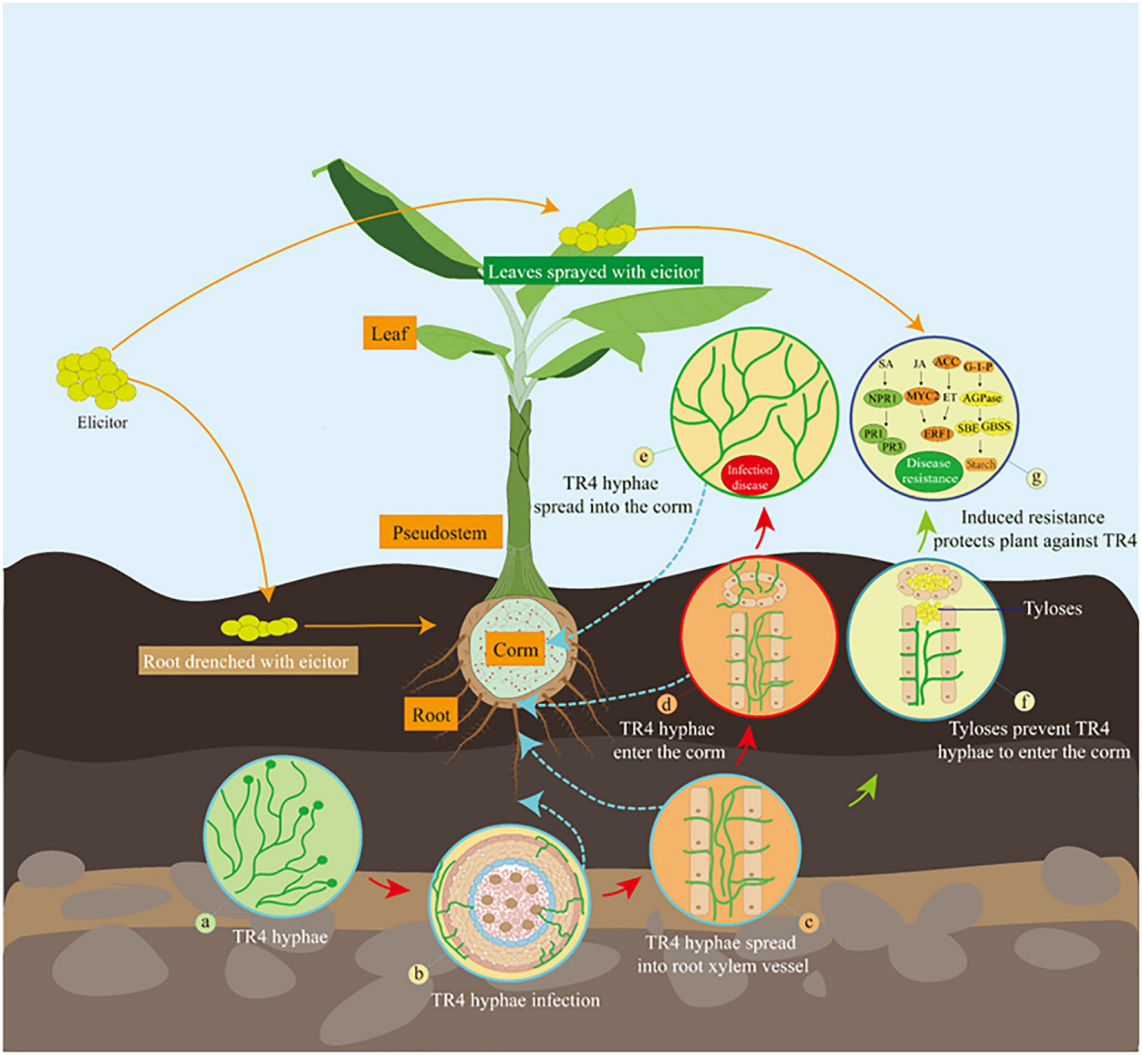
Scheme illustrating that isotianil-induced multi-resistance in bananas to prevent TR4 infection. **(A)** TR4 hyphae in the soil; **(B)** TR4 hyphae are accumulated in the rhizosphere and infestation into the plant; **(C)** TR4 hyphae enter plant root xylem vessel and spread further; **(D)** TR4 hyphae spread into the xylem vessel that connects the root to the corm; **(E)** TR4 hyphae spread into the corm and multiplies; **(F)** TR4 hyphae is blocked to enter the corm by elicitor -induced tyloses in the xylem vessel connecting the root to the corm; **(G)** After elicitor applicated plants by drenching roots or spraying leaves, multiple defense systems in the banana plant are activated to prevent further spread of TR4 in the corm. SA, salicylic acid; *NPR1*, nonexpressorofpathogenesis-relatedgenes1; *PR1*, pathogenesis-related 1 genes; *PR3*, pathogenesis-related 3 genes; JA, jasmonic acid; *MYC2*, basic helix-loop-helix transcription factor; *ERF1*, ethylene response factor 1; ET, ethylene; *ACC*, 1-aminocyclopropane-1-carboxylic acid synthase; *AGPase*, ADP-glucose pyrophosphorylase; SBE, Starch branching enzyme; *GBSS*, granule-binding starch synthase.

Isotianil is one such elicitors which acts as a salicylic acid (SA) mimic, with proven activity against rice blast (Bektas and Eulgem, 2014) and wheat blast (Portz et al., 2020). It was discovered by Bayer in 1997 (Toquin et al., 2012). Although isotianil does not have any direct antimicrobial activity against bacteria and fungi, it can induce the defense response of various plants to pathogens. For example, isotianil treatment can induce the expression of some defense-related genes, such as *NPR1* and *PR1* in the SA signaling pathway (Yoshida and Toda, 2013; Bektas and Eulgem, 2014). There is only one paten report on the application of isotianil in FWB before (Gilbert et al., 2019), and the specific mechanism of isotianil inducing plant resistance is also unclear yet. Therefore, this study aims to explore whether isotianil could induce plant resistance and alleviate the infection of FWB in different two cultivars. Besides, the interaction between TR4 and bananas have been explored by confocal laser-scanning microscope (CLSM) and molecular approaches have been used to analyze the mechanism of action mode of isotianil on banana plants (**Figure 1**).

## 2 Materials and methods

### 2.1 Plant materials

In this study, two cultivars of Cavendish were used, ‘Baxi’ (*Musa* spp. AAA, Susceptibility cultivar) and ‘Yunjiao No.1’ (*Musa* spp. AAA, moderately resistant cultivar), and the banana plantlets were propagated by plant tissue culture. The tissue plantlets were cultured at 25°C, under a 16 h/18 h (light/dark) photoperiod until new roots grew and then transplanted into an aperture disk (32 tray specification, capacity 110 mL), filled with coconut bran and seedling substrate. Each banana plantlets about 15 cm high with 5 leaves were transplanted into the plastic pot with 25 cm in the diameter containing garden soil and substrate. All banana plants were grown in a solar greenhouse with isotianil watering and fertilization management.

### 2.2 Isotianil application and pathogen inoculation

TR4 labeled with green fluorescent protein (GFP) was used to explore the infestation process of pathogens in plants (Zhang et al., 2018a). After TR4 was grown on PDA medium at 28 °C for 7 day, the spores were collected by rinsing the plates with sterile water, and the concentration of suspension was 1×10^7^ spores/mL measured by hemocytometer. Isotianil as the main compound of Routine^®^ product was provided by Bayer AG, Crop Science Division company. Routine^®^ is a suspension concentrate (SC) containing 0.2 g/mL isotianil and was applied when the banana plants had 6-7 leaves. The applied diluent was prepared by dissolving 0.035 mL of the original product in 100 mL of tap water per banana plant, applied by either drenching the roots or spraying the leaves (0.07 mg/mL isotianil, 100 mL/plant). The applications were performed once every 28 days, and the applications were performed three times in total. Seven days after the second application of Routine^®^, 100 mL of 1×10^7^ spores/mL of TR4 was drenched for root inoculation, and the control treatment was applied with tap water. Before TR4 inoculation, the roots were treated with two wounds around plants, using a shovel. Banana roots and corms were taken 0 d, 1 d, 7 d, 14 d, and 62 d after TR4 inoculation as samples for subsequent TR4 content detection, microscopic observation and gene expression determination.

### 2.3 Experimental treatment design

Four treatments were set of each banana cultivar which contain: control (CK), inoculated with pathogen alone (TR4), banana leaves sprayed Routine^®^ and inoculated with TR4 (TR4+R1) and Banana root drenched with Routine^®^ and inoculated with TR4 (TR4+R2) (**Table 1**). Three biological replicates were designed for per treatment and 45 plantlets were prepared for per replicate.

**Table 1:** Different treatments in pot experiment.

Treatment code	Cultivar	Description
CK	Baxi	Control
TR4	Baxi	Inoculated with pathogen TR4 alone
TR4+R1	Baxi	Banana leaves sprayed Routine^®^ and inoculated with TR4
TR4+R2	Baxi	Banana root drenched with Routine^®^ and inoculated with TR4
CK	Yunjiao No.1	Control
TR4	Yunjiao No.1	Inoculated with pathogen TR4 alone
TR4+R1	Yunjiao No.1	Banana leaves sprayed Routine^®^ and inoculated with TR4
TR4+R2	Yunjiao No.1	Banana root drenched with Routine^®^ and inoculated with TR4

### 2.4 Disease index investigation

The banana corms were dissected to investigate the disease index after 62 days post inoculated TR4. After plant corms were dissected, the degree of lesions of each plant corm was investigated according to five grades from 0 to 4. The five grades of 0, 1, 2, 3 and 4 represent no lesions of the corm, the area of corm lesions is 1-10%, the area of lesions in the corm is 11-30%, the area of corm lesions is 31-50% and the area of corm lesions is more than 50%, respectively. The formula for calculating the disease index and control effect is as follows (Zuo et al., 2018; Chen et al., 2019; Fan et al., 2021).


$$Disease\;index\;\left(\%\right)=\sum^{}\frac{Grade\times Number\;of\;plants\;in\;the\;grade}{4\times Total\;number\;of\;assessed\;plants}\times 100$$

$$Control\;effect\;\left(\%\right)=\frac{Control\;disease\;index-Treatment\;disease\;index}{Control\;disease\;index}\times 100$$

### 2.5 Detection of TR4 content in banana roots and corms by qPCR

The roots and corms of bananas plants after inoculation with TR4 were frozen in liquid nitrogen immediately after being collected at 4 different time points, and then stored at -80°C for later use. Genomic DNA was extracted according to the cetyltrimethylammonium bromide (CTAB) method (Tamari et al., 2013). Fungal biomass determination is basically genomic quantification of gene copy numbers by qPCR based on our previous established protocol (Zhang et al., 2018b). Three plants were prepared for each treatment as one biological replicate, and each treatment was repeated with three replicates. The quality of the resulting standard curve can be used for data analysis (efficiency, 90% to 110%; Correlation coefficient, R^2^>0.99).

### 2.6 Confocal laser scanning microscope observation

For examined infection process and colonization of TR4, the roots and corms of the banana plants were collected after inoculated with TR4. Three biological replicates were designed for per treatment and 3 plantlets were prepared for per replicate. The samples were washed in sterile water and 75% alcohol, and cut into transverse and longitudinal thin slices with an ultra-thin blade. The slices were placed on the microscope slide with MQ water droplets, and then cover the sample with a glass cover slip. The processed samples were microscopically observed under a confocal laser scanning microscope (Lecia TCS-SP, Wetzlar, Germany). The spectral parameters of GFP fluorescence and plant autofluorescence in this confocal laser scanning microscope are (excitation wavelength 488 nm, emission wavelength 500-560 nm) and (excitation wavelength 561 nm, emission wavelength 570-670 nm), respectively.

### 2.7 Determination of starch contents in corms

Fresh corms of different treatments were collected at 1 d, 7 d, 14 d and 62 d after inoculation TR4. Three plants were prepared for each treatment as one biological replicate, and each treatment was repeated with three replicates. The corms were frozen and ground into powder in liquid nitrogen immediately after collection, and the starch content was determined using the Plant Starch Content assay Kit (Comin Biotechnology Co Ltd. Suzhou, China) according to the manufacturer’s instructions.

### 2.8 Analysis of key genes expression related to starch synthesis and plant defense by quntitative real-time PCR

Three key genes (*AGPase*, *GBSS*, *SBE*) of banana in the starch synthetic pathway were selected for expression study at 1 d, 7 d, 14 d and 62 d post inoculation with TR4. Six defense-related gens *NPR1*, *PR1*, *PR3*, *MYC2*, *ERF1* and *ACC* were also selected for this study. In each treatment, 9 corms were collected to detect the expression of related genes, and three technical replicates and three biological replicates were performed for each analysis. The collected corms sample were immediately frozen in liquid nitrogen and stored at -80°C, and then the total RNA was extracted using the Omega Plant RNA Extraction Kit according to the manufacturer’s protocols. The A260/A280 and A260/A230 of total RNA were 1.9 to 2.1 and 2.0 to 2.4, respectively, and can be used for further experiments. Additionally, cDNA was synthesized by the Prime Script RT Master Mix Kit (TaKaRa), and Reverse-transcription quantitative PCR (RT-qPCR) was performed using the iTaq Universal SYBR Green Supermix Kit (BIO-RAD) according to the manufacturer’s protocols. Relative changes in gene expression levels were calculated by the 2^-△△^CT method (Zhao et al., 2013; Dong et al., 2019). Relevant primer sequences for RT-qPCR analysis are listed in **Supplementary Table 1** (Dong et al., 2019; Dalio et al., 2020). Moreover, *Musa25SrRNA* was used as the reference gene (Berg et al., 2007; Wu et al., 2013). In the preparation of the standard curve for real-time PCR amplification, each cDNA is diluted according to a gradient of 1-2-4-8-16-32-64-128, and then the corresponding standard curve is established. The R^2^ and amplification efficiencies of the standard curve were greater than 0.99 and between 90%-110%, respectively, and the next step of the experiment can be continued.

### 2.9 Data analysis

Data were analyzed by using SPSS 25 and were graphed using Origin2018 (Graph Pad Software). All values are expressed as mean ± standard deviation, and statistically significant differences were determined using the Duncan multiple range tests (*P<* 0.05).

## 3 Results

### 3.1 Isotianil application can significantly induce resistance of banana to Fusarium wilt

**Figure 2:**
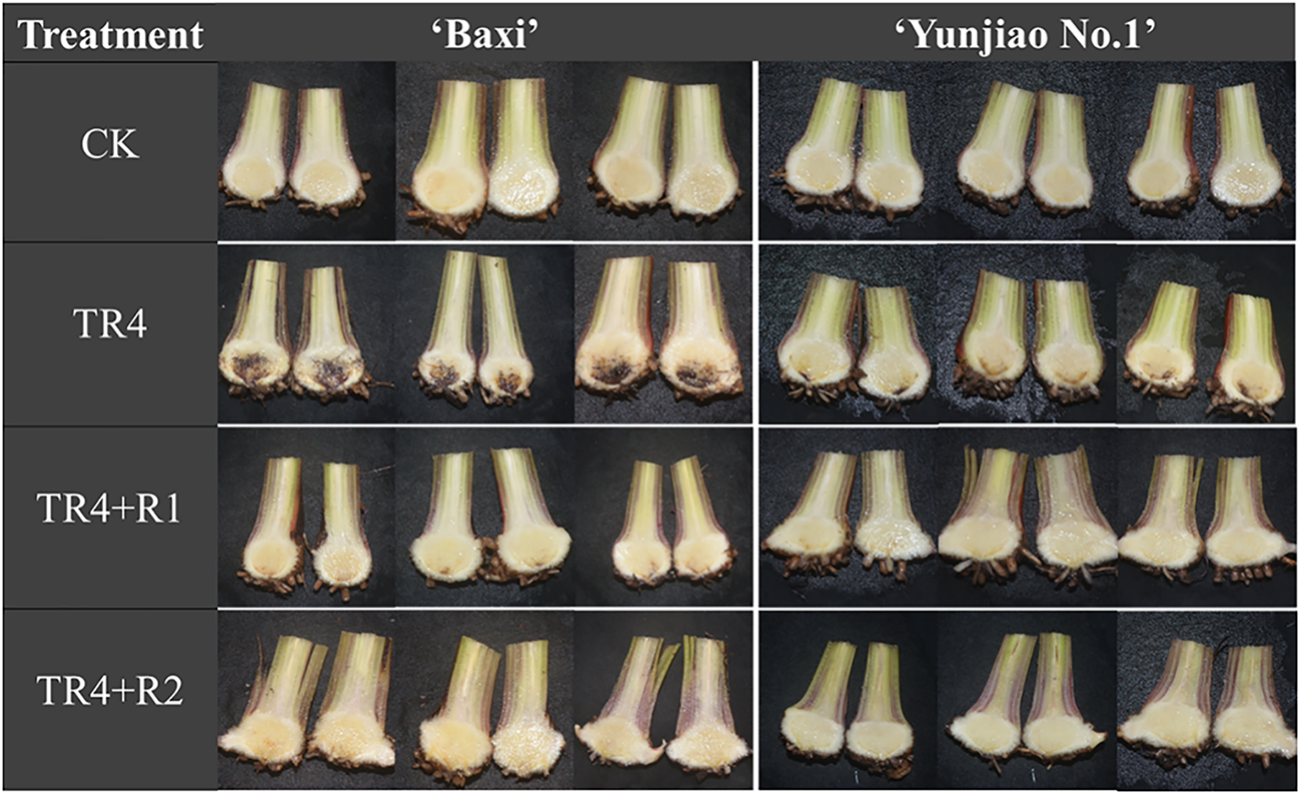
Corm dissection to show effect of isotianil application on banana plants after inoculated TR4. CK: control plants; TR4: plants inoculated with TR4; TR4+R1: plants applied with isotianil in leaves and inoculated with TR4; TR4 + R2: plants applied isotianil in roots and inoculated with TR4.

**Figure 3:**
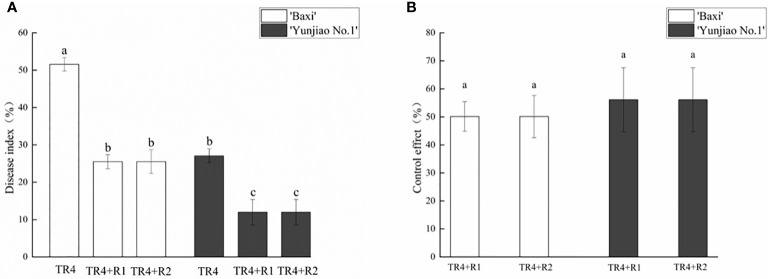
Effect of isotianil application on banana plants after inoculated TR4. **(A)** Disease index of all treatments. **(B)** Control effect of application with isotianil. The data represents three independently repeating values. Based on Duncan’s multiple range test, a significant difference was determined at P<0.05. Error bars represent ± standard deviation.

Banana corms were split at 62 dpi to investigate the disease severity, the symptoms of banana plants with TR4 infection were recorded. The untreated control treatment showed no symptoms or phytotoxicity. Compared with the control plants, the corms after TR4 inoculation showed obvious symptoms, and the color of the corms showing brownish-black zones of infection (**Figure 2**). However, the application of isotianil alleviated the symptoms caused by the TR4. Disease investigation showed that the disease indexes following isotianil application (‘Baxi’ 25.52% and ‘Yunjiao No.1’ 11.98%) were significantly lower than those where there was only TR4 inoculation (‘Baxi’ 51.56% and ‘Yunjiao No.1’ 27.08%) (**Figure 3A**). Among them, there was no significant difference between leaves sprayed (TR4+R1) or roots drenched (TR4+R2) with isotianil, but the disease indexes in ‘Yunjiao No.1’ were significantly lower than in ‘Baxi’. The control effects of isotianil in ‘Baxi’ and ‘Yunjiao No.1’ to FWB in greenhouse experiments were 50.14% and 56.14%, respectively (**Figure 3B**). For the control effects, there was no significant difference between leaves sprayed (TR4+R1) or roots drenched (TR4+R2) with isotianil. The results showed that ‘Yunjiao No.1’ is more resistant against TR4 than ‘Baxi’, and isotianil could significantly induce the resistance of both cultivars to FWB.

### 3.2 Determination of pathogen biomass in different tissues of banana plants

**Figure 4:**
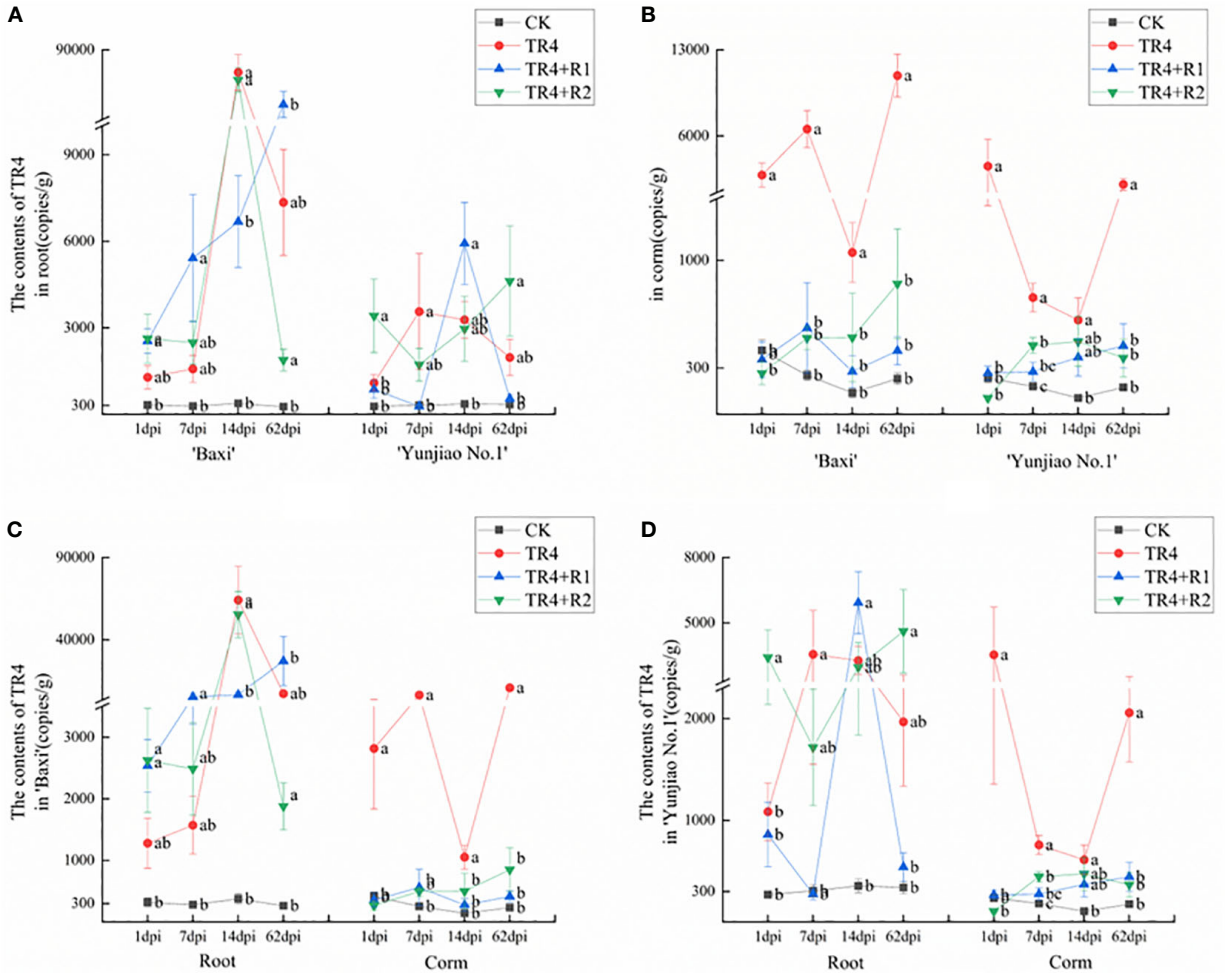
Biomass of TR4 in different tissue of banana cultivars at different time points after TR4 inoculation. **(A)** Differences of TR4 content in two cultivars in roots; **(B)** Differences of TR4 content in two cultivars in corms; **(C)** Differences of TR4 content in different tissue in ‘Baxi’; **(D)** Differences of TR4 content in different tissue in ‘Yunjiao No.1’. The data represents three independently repeating values. Based on Duncan’s multiple range test, a significant difference was determined at *P*<0.05. Lowercase letters a, b, c, d mark significant differences: the same letter means no difference during the same period, while different letters indicate a significant difference. Error bars represent ± standard deviation.

The pathogen biomass was measured by qPCR in different plant tissues at different time points (**Figure 4**). The results showed that the pathogen biomass of ‘Baxi’ (susceptible cultivar) was significantly higher than that of ‘Yunjiao No.1’ (resistant cultivar) in banana corms (**Figure 4B**). The pathogen biomass of corms in ‘Baxi’ and ‘Yunjiao No.1’ ranged from 139.58 ± 31.48 copies/g to 10905.68 ± 1745.75 copies/g and 104.65 ± 8.81 copies/g to 3540.11 ± 2184.47 copies/g, respectively. In addition, the content of TR4 in corms was significantly lower than root in all times points (**Figures 4C, D**). The pathogen biomass of corm and root in ‘Baxi’ ranged from 262.2 copies/g to 64,159.7 copies/g and 139.5 copies/g to 10,905.6 copies/g, respectively. There was no significant difference between TR4 inoculated plants (TR4), isotianil applied plants and inoculated with TR4 (TR4+R1, TR4+R2) in roots. However, the plants inoculated with TR4 (TR4) had significantly more pathogenic biomass than the control plants (CK) and the plants applied with isotianil and inoculated with TR4 (TR4+R1, TR4+R2) (**Figure 4B**).

### 3.3 Differences in the infection process of TR4 in banana plants

In order to explore the infection difference in different tissue, we used confocal laser-scanning microscope to monitor the infection colonization process of TR4 in banana tissue. The results showed that TR4 mainly existed in the form of hyphae in banana plants, and spores were hardly found. The detailed observation results were presented in **Supplementary Table 2**.

#### 3.3.1 TR4 infection in roots

**Figure 5:**
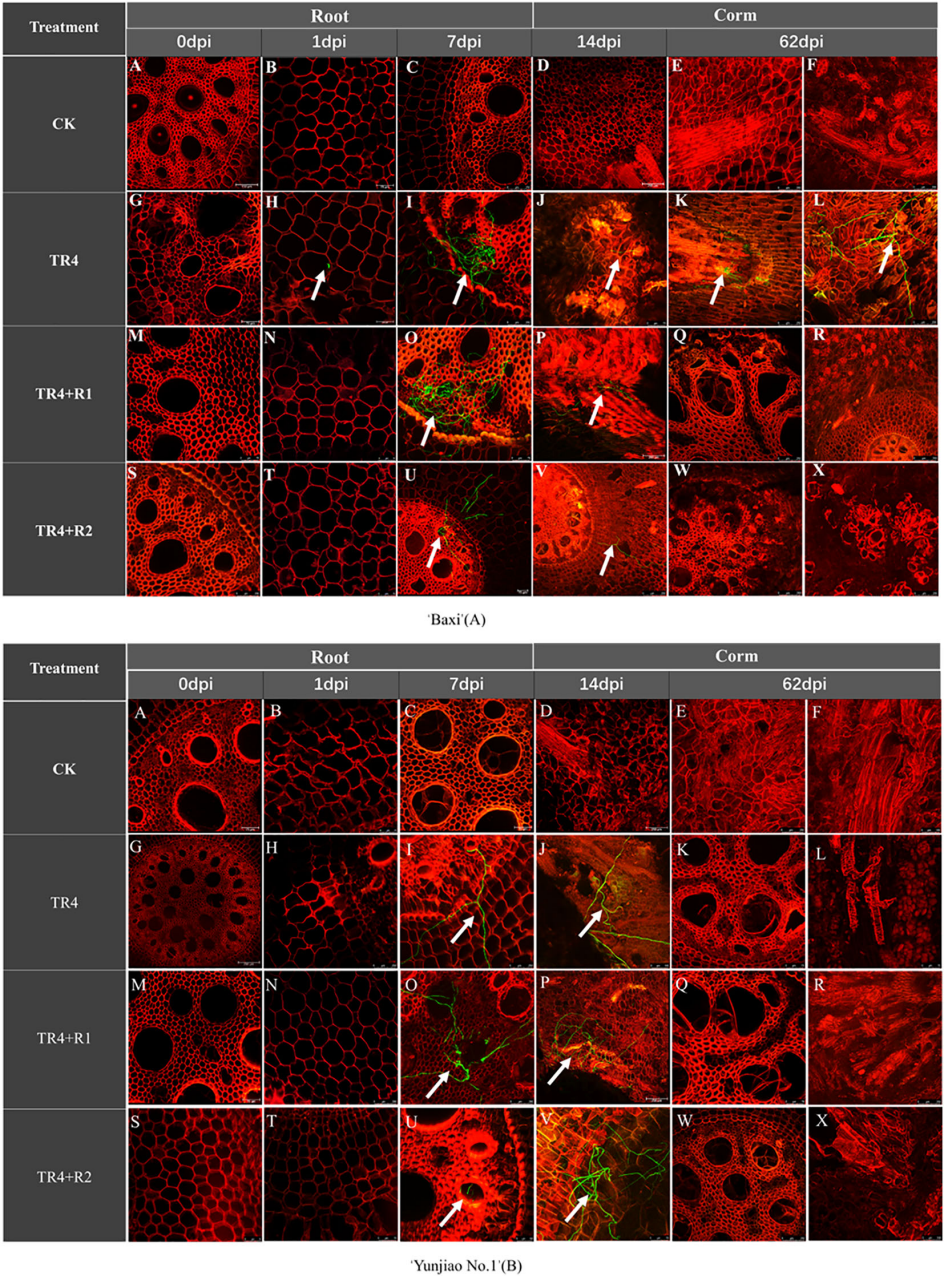
Hyphae expansion and infection in the root and corms of banana cultivar ‘Baxi’ inoculated with green fluorescent protein-tagged TR4 isolates at different time points (0 dpi, 1 dpi, 7 dpi, 14 dpi and 62 dpi). The TR4 hyphae was indicated by white arrows in the plant tissues. Photographs were taken under 488 (GFP) channel and 561 (RFP) channels (A-X). Bar = 250 μm in A, C, D, E, F, I, K, L, P, Q, R, S, U, V, W and X of ‘Baxi’ **(A)**; Bar = 75 μm in B, G, J, M, N, O and T of ‘Baxi’ **(A)**;Bar = 250 μm in D, E, G, H, I, N, O, P, R and W of ‘Yunjiao No.1’ **(B)**; Bar = 75 μm in A, B, C, F, J, K, L, M, Q, S, T, U, V and X of ‘Yunjiao No.1’ **(B)**.

The hyphae could penetrate the vascular bundle tissues of all treatments, but some differences were observed in their infection process. At 1 day post inoculation (dpi), the hyphae were observed in the vascular bundle tissues only in cultivar ‘Baxi’ inoculated TR4 treatments, but not observed in other treatments. At 7 dpi to 62 dpi, a large number of hyphae were observed in the vascular bundle of root and it multiplies in large numbers in all treatment (**Figure 5**).

#### 3.3.2 TR4 infection in corms

To accurately understand the infection mechanism of pathogens, TR4 hyphae in the corms from 1dpi to 62 dpi were further monitored. Few TR4 hyphae were discovered in the cortex vascular tissues at 7 dpi and then expanded to the central cylinder in cultivar ‘Baxi’ inoculated TR4 treatments at 14 dpi. However, hardly any TR4 hyphae were observed in the cortex and central cylinder in other treatments. At 14 dpi, a mass of hyphae was found in the cortical root vessels of corms in the cultivar ‘Baxi’ inoculated TR4 treatments, while relatively few hyphae were found in the cortical roots of the other treatments. At 62 dpi, massive hyphae were observed in vessels of corms in cultivar ‘Baxi’ inoculated with TR4 only, while the level of pathogen hyphae in other treatments was relatively rare, so we turned our attention to the central cylinder of corms again. During this period, the central cylinder of the cultivar ‘Baxi’ inoculated TR4 treatments was ruptured, and many TR4 hyphae were released from the ducts to colonize the central column of the corms, while in other treatments, no hyphae released from the central column were found (**Figure 5**).

### 3.4 Effect of isotianil application on TR4 infection between different cultivars and tissues

#### 3.4.1 Cultivar differences in TR4 infection between ‘Yunjiao No.1’ and ‘Baxi’

TR4 hyphae were observed in both roots and corms of ‘Baxi’ (TR4 inoculated plants) one day after inoculation with TR4, while were not observed in ‘Yunjiao No.1’. From 1dpi to 7dpi, the number of hyphae in the root and corms of ‘Yunjiao No.1’ is lower than that of ‘Baxi’. After 14 dpi, quite a lot of hyphae were discovered in the roots of ‘Baxi’ and ‘Yunjiao No.1’. A mass of hyphae was also discovered in the corms in ‘Baxi’, and almost no hyphae were observed in ‘Yunjiao No.1’. These results indicated that the infection difference of TR4 in ‘Yunjiao No.1’ and ‘Baxi’ was mainly in the corms, and ‘Yunjiao No.1’ was more resistant than ‘Baxi’. Furthermore, quantity of hyphae in the root is higher than in the corms. These results show that the corms play an important role in blocking the infection of TR4 (**Figure 5**).

#### 3.4.2 Effect of isotianil application on TR4 infection

One day after inoculated TR4, funguses were only discovered in the roots and corms of ‘Baxi’, but not in the tissues treated with isotianil. Numerous funguses were discovered in the roots and corms of all plants from 7dpi to 14dpi. However, a mass of funguses was discovered in the roots 62 dpi, while massively hyphae were observed only in the ‘Baxi’ without isotianil treatment in the corms, while ‘Yunjiao No.1’ and ‘Baxi’ treated with isotianil no mycelia were observed (**Figure 5**) According to the tracking of the TR4 infection process over a period of time and across different plant parts, these results show that isotianil application can trigger the resistance of banana plants and prevent TR4 hyphae from infecting corms.

### 3.5 Expression of genes related to plant defense in bananas

The relative expression of genes *NPR1, PR1, PR3*, *MYC2*, *ERF1* and *ACC* in corms was analyzed in the control (CK), TR4 inoculated plants (TR4) and isotianil treatment with TR4 inoculated plants (TR4+R1, TR4+R2). In both cultivars, isotianil application significantly induced or repressed the expression of *PR1*, *PR3* and *NPR1* in the pathways regulating salicylic acid at different time point. Compared with TR4 treatment alone, the expression of *PR1* gene was significantly upregulated by routine application in ‘Baxi’ at different time point. The gene expression levels of TR4 inoculated plants (TR4) and isotianil application with TR4 inoculated plants (TR4+R1, TR4+R2) were as TR4-1 dpi: 4.70 ± 0.88; TR4-7 dpi: 0.36 ± 0.20; TR4-14 dpi: 7.83 ± 1.56; TR4+R1-1 dpi: 32.79 ± 7.50;TR4+R2-1 dpi: 38.87 ± 8.86; TR4+R1-7 dpi: 7.71 ± 2.46; TR4+R2-7 dpi: 5.14 ± 2.20;TR4+R-14 dpi: 19.47 ± 2.23; TR4+R2-14 dpi: 29.96 ± 9.44, respectively. In ‘Yunjiao No.1’, the *PR1* gene expression levels of isotianil application with TR4 inoculated plants (TR4+R1, TR4+R2) were as TR4+R1-7 dpi: 8.83 ± 1.37; TR4+R2-7 dpi: 4.28 ± 0.81; TR4+R1-14 dpi: 3.27 ± 0.66; TR4+R1-62 dpi: 5.34 ± 1.41; TR4+R2-62 dpi: 3.14 ± 1.02, which were significantly upregulated than those of inoculation with TR4 alone (TR4-7 dpi:1.12 ± 0.19; TR4-14 dpi:2.4 ± 0.78; TR4-62 dpi:2.56 ± 0.55), respectively. Additionally, compared with the TR4 treatment alone (TR4), the *NPR1* gene expression of ‘Yunjiao No.1’ was significantly upregulated by isotianil application (TR4+R1 and TR4+R2), and the expression levels of were as TR4-7 dpi: 0.62 ± 0.09; TR4-14 dpi: 0.37± 0.09; TR4-62 dpi: 0.45 ± 0.14; TR4+R2-7 dpi: 1.22 ± 0.17; TR4+R1-14 dpi:1.28 ± 0.24; TR4+R2-14 dpi: 1.21 ± 0.45; TR4+R1-62 dpi: 1.11 ± 0.39; TR4+R2-62 dpi: 1.34 ± 0.54, respectively. In addition, the genes *ERF1* that ISR-related genes were upregulated and downregulated as a result of the isotianil application in ‘Yunjiao No.1’ and ‘Baxi’, respectively. In ‘Yunjiao No.1’, the *ERF1* gene expression levels of routine application were as TR4+R1-7 dpi: 9.68 ± 1.48; TR4+R2-7 dpi: 36.60 ± 6.65; TR4+R1-14 dpi: 4.45 ± 0.38; TR4+R2-14 dpi: 1.85 ± 0.29; TR4+R1-62 dpi: 76.05 ± 26.04, which were significantly upregulated than those of inoculation with TR4 alone (TR4-7 dpi:2.23 ± 0.16; TR4-14 dpi:0.26 ± 0.11; TR4-62 dpi:3.32 ± 1.06), respectively. Additionally, the *ERF1* gene expression of ‘Baxi’ were no significantly in TR4 treatment alone (TR4) and isotianil application treatment. Compared with the control treatment (CK), the *ACC and MYC2* genes expression of ‘Yunjiao No.1’ were significantly upregulated by TR4 (TR4), and the expression levels of *ACC* and *MYC2* were as TR4-7 dpi: 0.76 ± 0.09; TR4-14 dpi: 0.64± 0.13; TR4-62 dpi: 0.45 ± 0.08; TR4-1 dpi: 0.27 ± 0.03; TR4-7 dpi:0.45 ± 0.05; TR4-14 dpi: 0.73 ± 0.31; TR4-62 dpi: 0.25 ± 0.07, respectively, while there was no obvious trend in ‘Baxi’. All detailed results were presented in **Supplementary Table 3**.

### 3.6 Tyloses accumulation in the vascular bundles of corms

**Figure 6:**
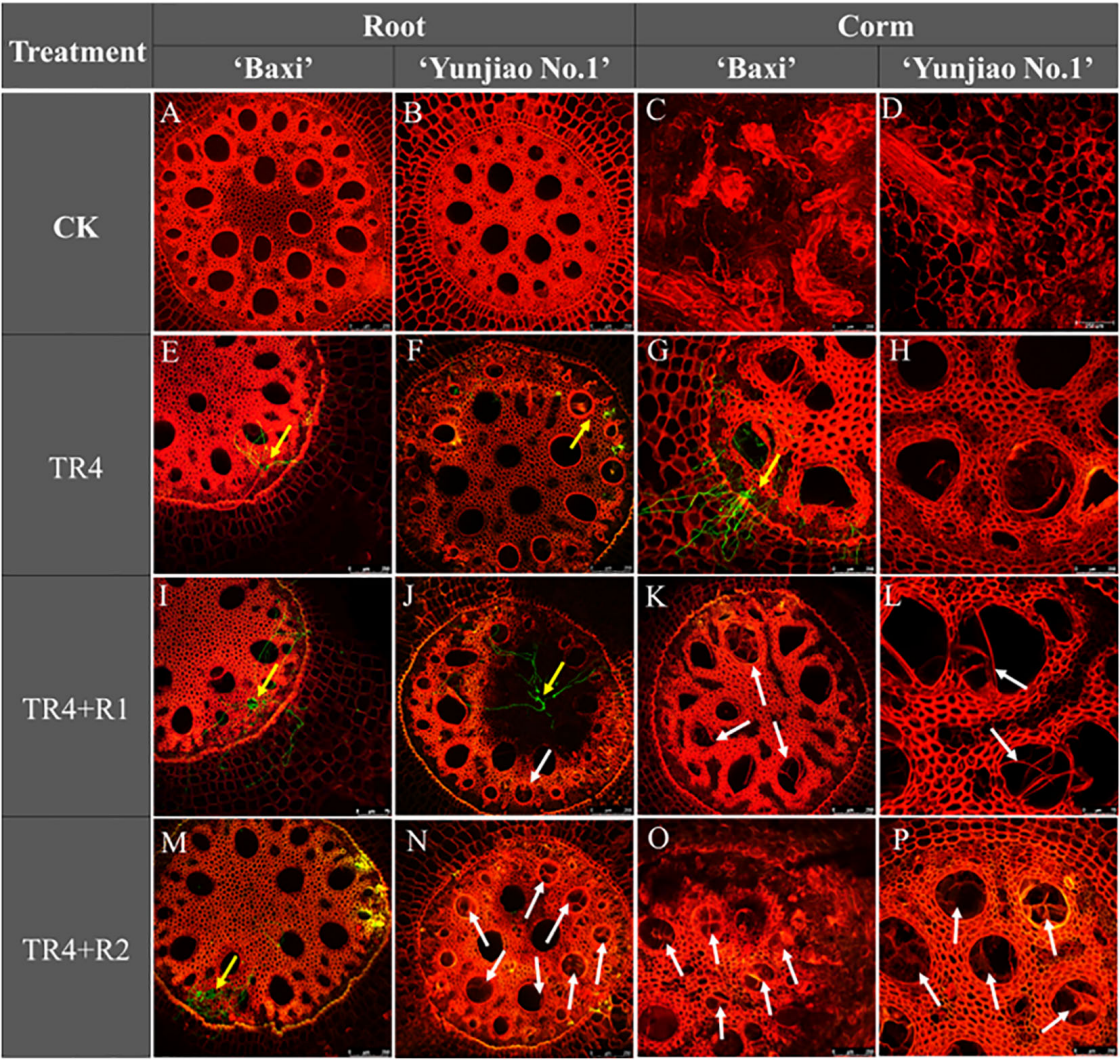
Microscopic observation of tyloses in banana corms and root at 62 days post inoculation TR4 (62 dpi) and mock inoculation. The tyloses was indicated by white arrows in the banana plant tissues. The TR4 hyphae was indicated by yellow arrows in the banana plant tissues. Photographs were taken under 488 (GFP) channel and 561 (RFP) channels **(A–P)**. Bar = 250 μm in **(A–H, J, K, M–P)**; Bar = 75 μm in I.

A microscopic observation analysis performed on root and corms samples from banana plants showed that tyloses plays an important role in against the pathogen infection in vascular bundle. At 62 days post inoculation (dpi), numerous tyloses were observed in the cortical root vascular bundle vessels of corms. At the same time, the hyphae were significantly reduced in the tissues with tyloses (**Figures 6I–P**) whereas a large number of TR4 hyphae were observed in the tissues without tyloses (**Figures 6E–M**). Isotianil application could induce the formation of tyloses (**Figures 6J–P**) and the number of tyloses of isotianil treatments (TR4+R1, TR4+R2) were higher than that in TR4 infected plants (TR4). There were also differences between different banana cultivars, with the tyloses in ‘Yunjiao No.1’ were also being more abundant than in ‘Baxi’.

### 3.7 Determination of starch content and related gene expression levels in corms

**Figure 7:**
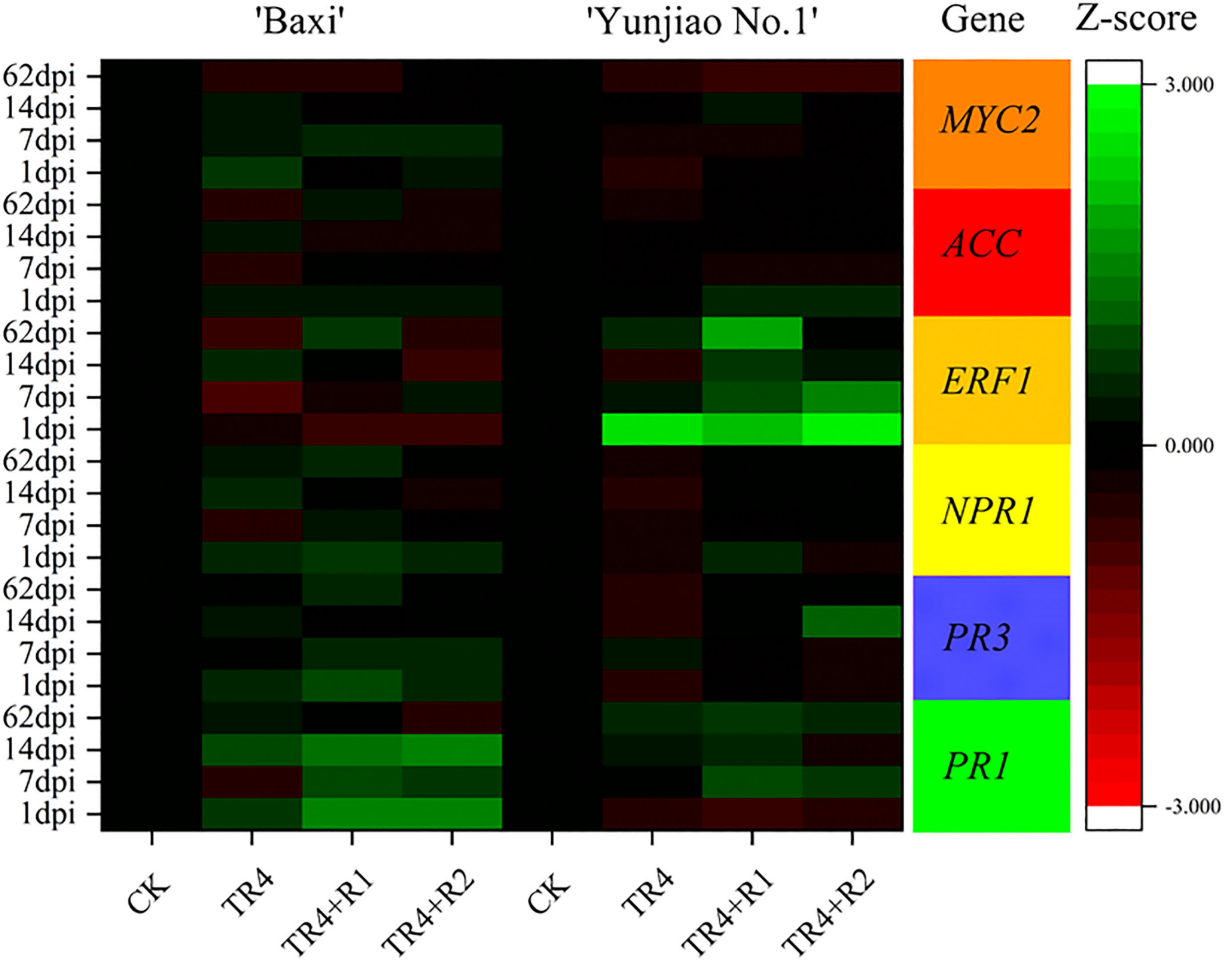
Relative expression levels of key genes that induce resistance in corms at 1 dpi to 62 dpi. The heat map illustrates the doubled changes of gene expression (log10 scale) in corms at 1 dpi, 7 dpi, 14 dpi and 62 dpi. Different color means induced or repressed gene expression (Red indicates down-regulation of gene expression; green indicates up-regulation of gene expression; black indicates no effect on gene expression). Three biological replicates and three technical replicates were used in data analysis. MYC2, basic helix-loop-helix (bHLH) transcription factor; ACC, 1-aminocyclopropane-1-carboxylic acid synthase; ERF1, ethylene response factor 1; NPR1, nonexpressorofpathogenesis-relatedgenes1; PR3, pathogenesis-related 3 genes; PR1, pathogenesis-related 1 genes.

In the process of monitoring TR4 infection, there were significantly less TR4 hyphae in cells filled with starch granules than in tissues with fewer starch granules. In addition, the content of starch granules at 62 dpi was higher than that at 1dpi(**Supplementary Figures 1A, B**). These results indicated that starch granules in corms may play an important role in preventing TR4 infection (**Figure 7**). To verify that starch content is related to plant disease resistance, the total starch content in the bulbs was determined (**Figure 7A**). The results showed that the starch contents in the isotianil applied banana plants (TR4+R1, TR4+R2) were significantly higher than those in TR4 inoculation alone (TR4) and control (CK) in ‘Baxi’. In addition, the starch content in ‘Yunjiao No.1’ is significantly higher than that in ‘Baxi’. The starch content of corm in ‘Baxi’ and ‘Yunjiao No.1’ ranged from 15.25 ± 3.18 mg/g to 67.01 ± 4.39 mg/g and 44.70 ± 0.73 mg/g to 123.05 ± 10.89 mg/g, respectively (**Figure 7A**). These results indicate that isotianil applied plants were induced to produce more starch granules than TR4 inoculated plants (**Figure 7**). In addition, the key genes (*SBE*, *GBSS*, *AGPase*) related to starch synthesis in the corm were selected for its expression study, and the results showed that the expression of *SBE*, *GBSS* and *AGPase* in two cultivars was significantly different. These genes are significantly more expressed in ‘Yunjiao No.1’ than ‘Baxi’ (**Figure 7B**).

## 4 Discussion

In the past few decades, the banana production suffered dramatic losses because of the epidemic of FWB, which is a typical soil-borne disease that is difficult to control (Ortiz, 2013; Zhang et al., 2013; Li et al., 2015; Wang et al., 2015; Paz‐Ferreiro and Fu, 2016; Zuo et al., 2018; Niwas et al., 2020). According to the historical experience of the first epidemic of FWB, enhancing plant disease resistance is generally considered to be one of the most effective strategies to control FWB. However, the mechanism by which banana plants resist TR4 infection is unclear.

From this study, the results showed that isotianil can significantly reduce the incidence of FWB and alleviate the disease symptoms in both cultivars (**Figures 2**, **3**). Comparing the disease index, the isotianil application treatments (TR4+R1, TR4+R2) was significantly lower than that TR4 inoculated alone (TR4). To explain the mechanism of action of isotianil on banana plants, the biomass of TR4 in different banana tissues were measured by qPCR. The results showed that TR4 biomass in corms is lower after isotianil application, and both cultivars have a consistent trend (**Figure 4B**). The TR4 biomass in the corm was consistent and stable at different growth points. However, no clear tendency of TR4 biomass in roots was found, probably because roots keep growing and only parts of roots were taken for the analysis; sampled roots could be newly grown and uninfected. At the same time, qPCR results showed that at 14 dpi, the content of the pathogenic in corms alone TR4 inoculation gradually decreased, which may be related to the increase of plant resistance during this period. In addition, TR4 biomass in corms was significantly lower than in roots, and these results were consistent with what was observed. Therefore, these results showed that the corm as a physical barrier can play an important role in reducing the harm of FWB by slowing down TR4 infestation. This result is consistent with our previous study (Zhang et al., 2018b). However, the detailed mechanism which prevents TR4 entering the corm is deserved for further study. In the current study, in order to make it easier to monitor the infestation process of TR4 in bananas plants, *GFP*-TR4 was used (Zhang et al., 2018a). The results showed that TR4 hyphae penetrates the root epidermis, invades the xylem vessels, and also invades the vascular bundle vessels from the wound, root hair or intercellular spaces of the cortex. These phenomena are basically consistent with previous research results (Li et al., 2011; Guo et al., 2014; Guo et al., 2015; Li et al., 2017). Compared with other parts during the infection process, we found that TR4 hyphae were more likely to infect from wounds, which is similar to the findings of Dong (Dong et al., 2019). Study of the TR4 infection process in the past was mainly focused on the infection of plant roots or corms (Li et al., 2011; Guo et al., 2015; Li et al., 2017). However, no one observed how TR4 moves from the roots into the corms. In this study, we observed that TR4 hyphae enter the corms from the root through vascular ducts, and the number of TR4 hyphae in the root is much higher than in the corms. Once again, these results confirmed that banana corms play an important role for blocking the infection of pathogens. At the same time, the observation by confocal laser scanning microscope showed that the number of TR4 hyphae after isotianil application corms (TR4+R1, TR4+R2) was significantly lower than that in TR4 treatment (TR4). When plants were infected by pathogens, the defense response in the xylem vessels would be activated, preventing further spread of the pathogens (Yadeta and Thomma, 2013; Li et al., 2022). One of the common defense mechanisms produced in xylem vessels is the formation of tyloses (Beckman, 1964; Talboys, 1972; Grimault et al., 1994; Rahman et al., 1999; Fradin and Thomma, 2006; Yadeta and Thomma, 2013). The formation time and number of tyloses in different plants varied greatly, and the contents of tyloses in disease-resistant plants were significantly higher than in disease-susceptible plants (Grimault et al., 1994; Xu et al., 1997; Fradin and Thomma, 2006; Hu et al., 2008).In this study, a mass of tyloses and gums were observed in the cortical roots of banana plants treated with isotianil, while almost no pathogen was observed where the tyloses appeared (**Figure 6**). Therefore, this result showed that isotianil could induce the formation of tyloses in the root of banana cortex to prevent the further infection of TR4 into corms.

**Figure 8:**
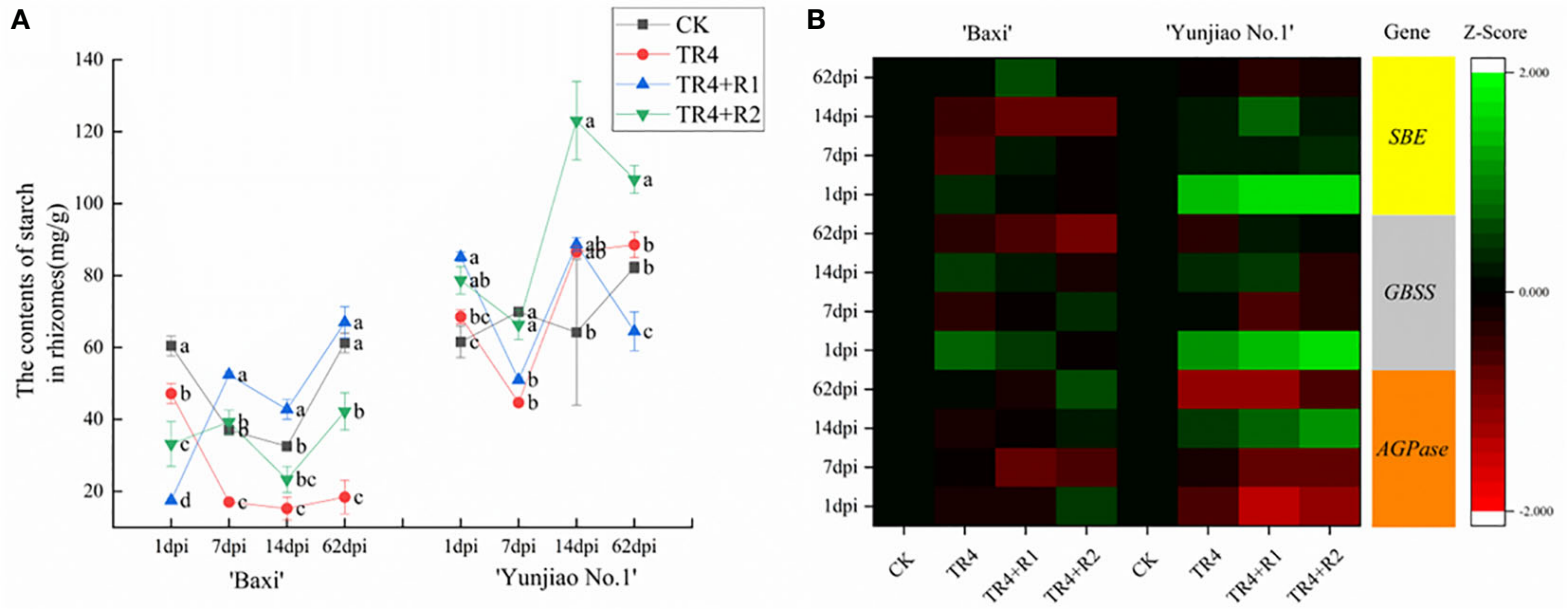
**(A)** Contents of starch in banana corms at different time points past inoculation TR4 (1 dpi, 7 dpi, 14 dpi and 62 dpi). The data represents three independently repeating values. Based on Duncan’s multiple range test, a significant difference was determined at P<0.05. Lowercase letters a, b, c, d mark significant differences: the same letter means no difference during the same period, while different letters indicate a significant difference. Error bars represent ± standard deviation. **(B)** Relative expression levels of key genes for starch synthesis in corms at 1dpi to 62dpi. The heat map illustrates the double change in the expression(log10) of key genes for starch synthesis in the corm at 1dpi to 62 dpi. Different color means induced or repressed gene expression (Red indicates down-regulation of gene expression; green indicates up-regulation of gene expression; black indicates no effect on gene expression). Three biological replicates and three technical replicates were used in data analysis. AGPase, ADP-glucose pyrophosphorylase; SBE, Starch branching enzyme; GBSS, granule-binding starch synthase.

When plants encounter some stress challenges, they can quickly initiate corresponding defense responses to enhance their resistance (Conrath et al., 2002; Acharya et al., 2011; Tanou et al., 2012). Elicitors are a class of substances that can trigger defense responses by mimicking the interaction of corresponding signaling molecules with homologous receptors in plants (Nimchuk et al., 2003). In the study, prior applications of isotianil significantly enhanced the expression of *PR1*, *PR3*, *NPR1* and *ERF1* and further increased the expression in isotianil application plants (TR4+R1 and TR4 +R2) when compared to TR4 inoculated plants. Isotianil pre-application significantly induced the expression of key genes *PR1*, *PR3* and *NPR1* of the SA pathway in banana plants. The result showed that isotianil may initiate the SA pathway to improve banana resistance to Fusarium wilt (**Figure 8**), this is consistent with previous research results on rice (Toquin et al., 2012). Some studies have shown that ERF1, a key responder downstream of the ET and JA pathway (Zhu et al., 2011; Huang et al., 2016), plays an important role in plant disease resistance (Berrocal-Lobo and Molina, 2004; Meng et al., 2013; Xing et al., 2017). In isotianil application plants, ISR-related genes were also induced to significantly upregulate at some time points, such as *ERF1* (**Figure 8**), this part of the result is a new discovery. Taken together, these results suggest that isotianil is a potent resistance inducer that can significantly enhance the expression of key genes of SAR and ISR pathways in banana plants (**Figure 8**).

Another interesting finding was that there was a large accumulation of starch grains in the corm cells (**Supplementary Figure 1**), and starch granules and TR4 in diseased corms could not coexist in the same time and space (Dong et al., 2019). At the same time, many studies have reported that under abiotic stress, the starch content will decrease or increase (Villadsen et al., 2005; Pressel et al., 2006; Goyal, 2007; Damour et al., 2008). The accumulation of starch granules is not only an important factor for plants to respond to abiotic stress, but also closely related to plant disease response (Takushi et al., 2007; Etxeberria et al., 2009). Combined with the previous observations, the expression of starch synthesis-related genes in corm was measured by qPCR, and the results showed that the expression of key genes in ‘Yunjiao No.1’was significantly upregulated, and the upregulation level was higher after isotianil application. (**Figure 7B**). In addition, the expression of related genes in ‘Yunjiao No.1’ at some time point was much higher than in ‘Baxi’. Further, the starch content in corms of different cultivars was measured, and the results showed that in ‘Yunjiao No.1’ (disease-resistant cultivar) the level was much higher than that in ‘Baxi’ (susceptible cultivar). In addition, this result showed that the starch content after the isotianil application was much higher in ‘Baxi’ (**Figure 7A**). On the one hand, the accumulation of starch may enhance the density of cell (Kuang et al., 2013) and directly inhibit the diffusion of TR4 in the corm, on the other hand, starch, as an important energy substance, may directly participate in the synthesis of resistance-related substances in cells (Dong et al., 2019). Therefore, we speculate that the accumulation of starch grains in the corm cells may be closely related to the disease resistance of plants.

There are differences in the resistance of two cultivars. Our results showed that ‘Yunjiao No.1’ can significantly reduce the incidence of FWB and alleviate the disease symptoms much more than ‘Baxi’ cultivar. Comparing ‘Baxi’ with ‘Yunjiao No.1’, the disease index of ‘Yunjiao No.1’ was significantly lower than that of ‘Baxi’ (**Figure 3**). In addition, the TR4 biomass in ‘Yunjiao No.1’ (2058.42 copies/g) is much lower than that of ‘Baxi’ (10905.69 copies/g) in corms at 62 dpi (**Figure 4B**). Moreover, the content of tyloses and starch grains in ‘Yunjiao No.1’ is higher than that of ‘Baxi’, and there is a consistent trend of disease resistance between ‘Yunjiao No.1’ and ‘Baxi’ applicated with isotianil. Furthermore, the expression of resistance (*ERF1*) and starch related (*AGPase*, *SBE* and *GBSS*) genes in ‘Yunjiao No.1’ are higher than those in ‘Baxi’. All the results demonstrated that ‘Yunjiao No.1’ is more resistant to FWB than ‘Baxi’, and resistant banana plants may prevent pathogenic fungal infection by inducing the content of tyloses in vascular bundles and starch grains in corms to form a physical barrier and activate immune pathways such as SAR and ISR in the corms.

In the next step of our research, we will continue to explore the defense mechanisms of different resistant banana cultivars in the face of TR4 infestation, especially the relationship between tyloses, starch grain formation and plant resistance.

## 5 Conclusion

This study has shown that the plant elicitor isotianil can significantly reduce the impact of FWB and protect different banana cultivars. In addition, we further found that corms are important to against further infestation of TR4. The elicitor isotianil is able to induce the formation of tyloses in cortex vascular tissues of corms, preventing pathogen from root entering the corms. It can activate the three major systems of ISR, SAR, and starch granule synthesis in the corms, and inhibit the diffusion of pathogen in the corms, so as to reduce the effect of FWB. In addition, ‘Yunjiao No.1’ are more resistant than ‘Baxi’. In summary, when biological, chemical and agronomic measures are not ideal for the control of FWB, further enhance the resistance of cultivar together with elicitor isotianil application is a promising control strategy for banana growers.

## Data availability statement

The original contributions presented in the study are included in the article/**Supplementary Material**. Further inquiries can be directed to the corresponding authors.

## Author contributions

Conceptualization, G-DZ, SL and S-JZ. Methodology and software, G-DZ, PH and SL. Validation, G-DZ, PH, SL and S-JZ. Performed the experiment and data analysis, LT, SX, BY, LL, YW, TB and XL. Writing—original draft preparation, G-DZ, PH and SL. Writing—review and editing, SL and S-JZ. Supervision and project administration, SL and S-JZ. Project administration and funding acquisition, S-JZ. All authors have read and agreed to the published version of the manuscript.

## Supplementary material

Supplementary Figure 1 Observation of starch granules in corms cell at different time points (1 dpi, 7 dpi, 14 dpi and 62 dpi) after inoculation TR4. The TR4 hyphae and starch grains was indicated by white arrows and yellow arrows respectively in the banana plant corms. Photographs were taken under GFP channel, GFP channel and through transmitted light (A-P). bar = 250 μm in A, B, C, D, E, G, H, J, K, L, M, O and P of ‘Baxi’ **(A)**; bar = 75 μm in F, I, and N of ‘Baxi’ **(A)**; Bar = 250μm in A, B, C, D, E, F, H, K, L, N, O and P of ‘Yunjiao No.1’ **(B)**; bar = 75 μm in G, I, J and M of ‘Yunjiao No.1’ **(B)**.
