# Supplementary figures and images for: Disentangling the resistant mechanism of Fusarium wilt TR4 interactions with different cultivars and its elicitor application
Source: Front Plant Sci. 2023 Mar 2;14:1145837. doi: 10.3389/fpls.2023.1145837 (PMC10018200; doi:10.3389/fpls.2023.1145837)

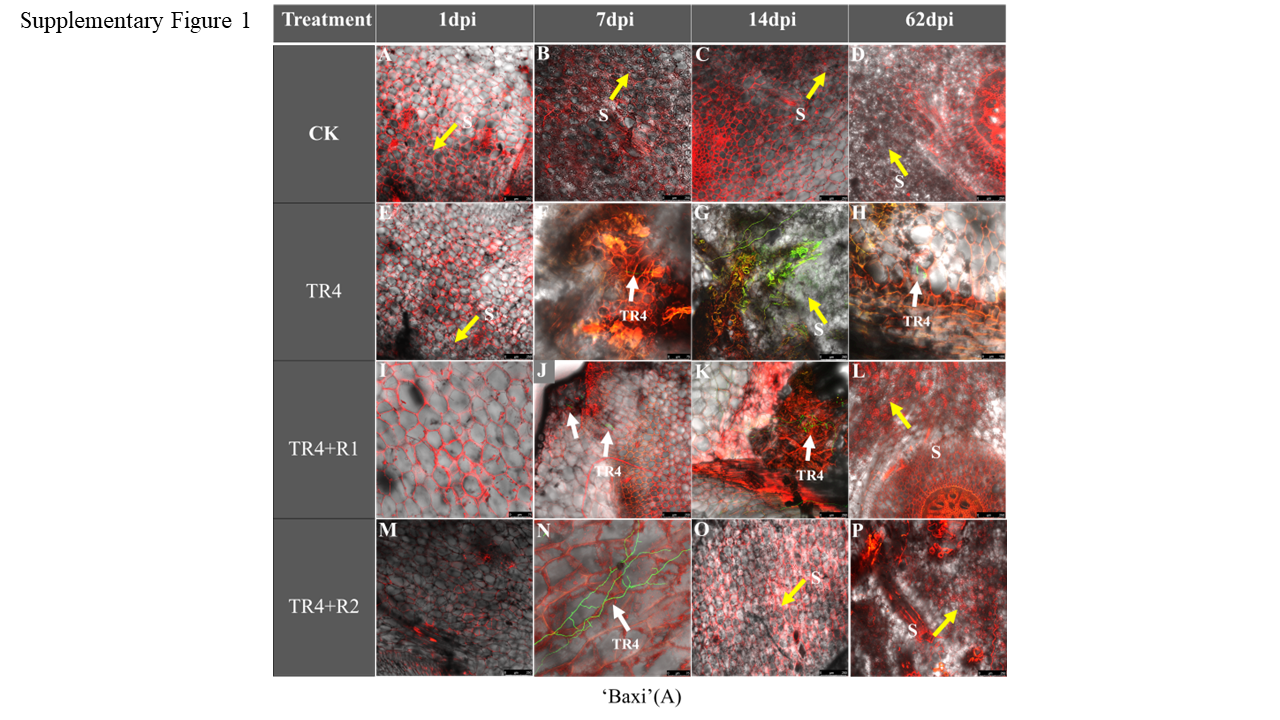

Supplement: Supplementary Figure 1 — Observation of starch granules in corms cell at different time points (1 dpi, 7 dpi, 14 dpi and 62 dpi) after inoculation TR4. The TR4 hyphae and starch grains was indicated by white arrows and yellow arrows respectively in the banana plant corms. Photographs were taken under GFP channel, GFP channel and through transmitted light (A-P). bar = 250 μm in A, B, C, D, E, G, H, J, K, L, M, O and P of ‘Baxi’ (A); bar = 75 μm in F, I, and N of ‘Baxi’ (A); Bar = 250μm in A, B, C, D, E, F, H, K, L, N, O and P of ‘Yunjiao No.1’ (B); bar = 75 μm in G, I, J and M of ‘Yunjiao No.1’ (B). [file Image_1.tif]

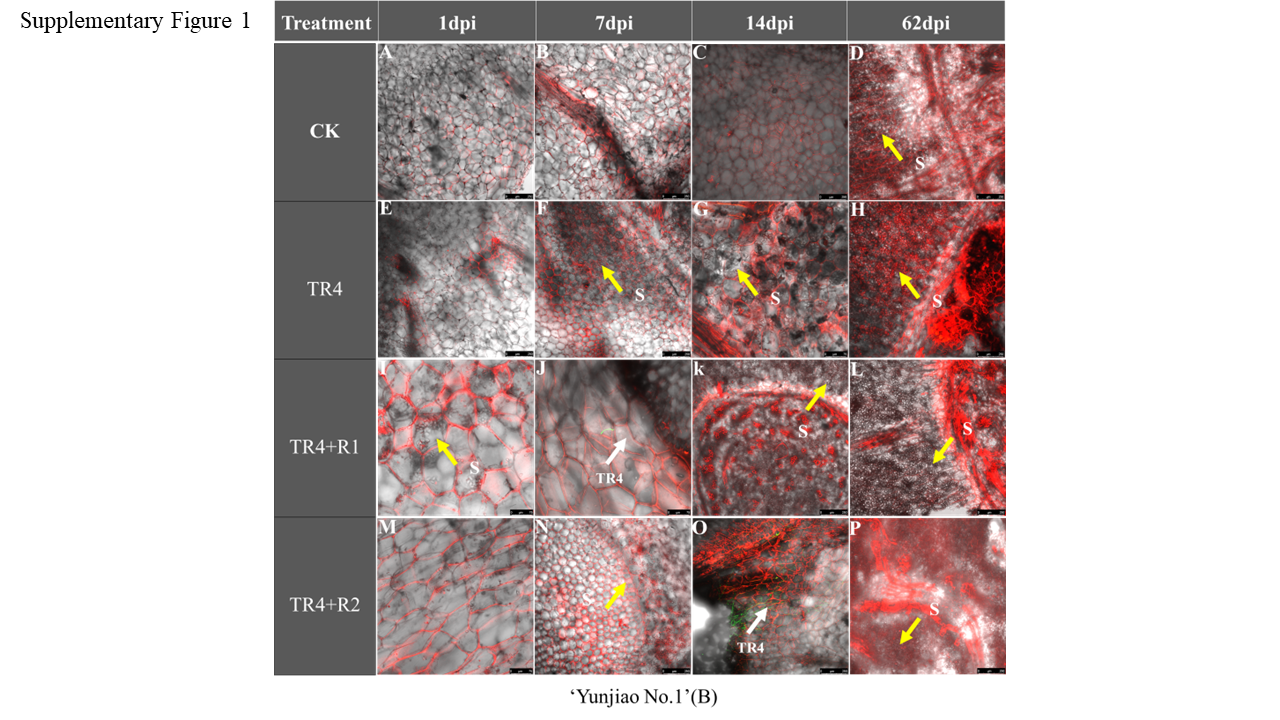

Supplement: Supplementary file 2 [file Image_2.tif]
