# Supplementary material for: Disentangling the resistant mechanism of Fusarium wilt TR4 interactions with different cultivars and its elicitor application
Source: Front Plant Sci. 2023 Mar 2;14:1145837. doi: 10.3389/fpls.2023.1145837 (PMC10018200; doi:10.3389/fpls.2023.1145837)
Supplement: Supplementary file 3 [file Presentation_1.pptx]

## Slide 1
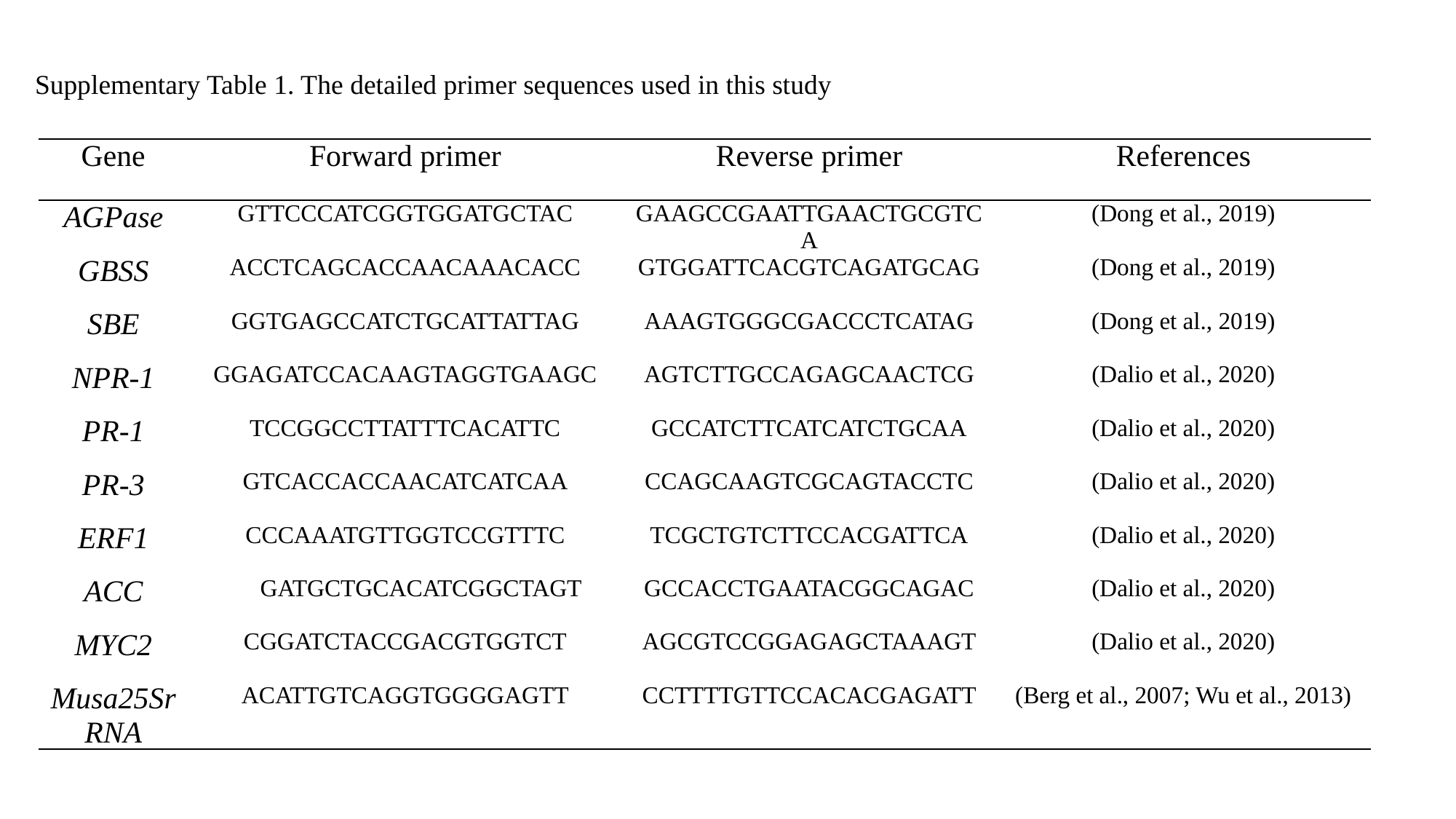

Supplementary Table 1. The detailed primer sequences used in this study
| Gene | Forward primer | Reverse primer | References |
| --- | --- | --- | --- |
| AGPase | GTTCCCATCGGTGGATGCTAC | GAAGCCGAATTGAACTGCGTCA | (Dong et al., 2019) |
| GBSS | ACCTCAGCACCAACAAACACC | GTGGATTCACGTCAGATGCAG | (Dong et al., 2019) |
| SBE | GGTGAGCCATCTGCATTATTAG | AAAGTGGGCGACCCTCATAG | (Dong et al., 2019) |
| NPR-1 | GGAGATCCACAAGTAGGTGAAGC | AGTCTTGCCAGAGCAACTCG | (Dalio et al., 2020) |
| PR-1 | TCCGGCCTTATTTCACATTC | GCCATCTTCATCATCTGCAA | (Dalio et al., 2020) |
| PR-3 | GTCACCACCAACATCATCAA | CCAGCAAGTCGCAGTACCTC | (Dalio et al., 2020) |
| ERF1 | CCCAAATGTTGGTCCGTTTC | TCGCTGTCTTCCACGATTCA | (Dalio et al., 2020) |
| ACC | GATGCTGCACATCGGCTAGT | GCCACCTGAATACGGCAGAC | (Dalio et al., 2020) |
| MYC2 | CGGATCTACCGACGTGGTCT | AGCGTCCGGAGAGCTAAAGT | (Dalio et al., 2020) |
| Musa25SrRNA | ACATTGTCAGGTGGGGAGTT | CCTTTTGTTCCACACGAGATT | (Berg et al., 2007; Wu et al., 2013) |

## Slide 2
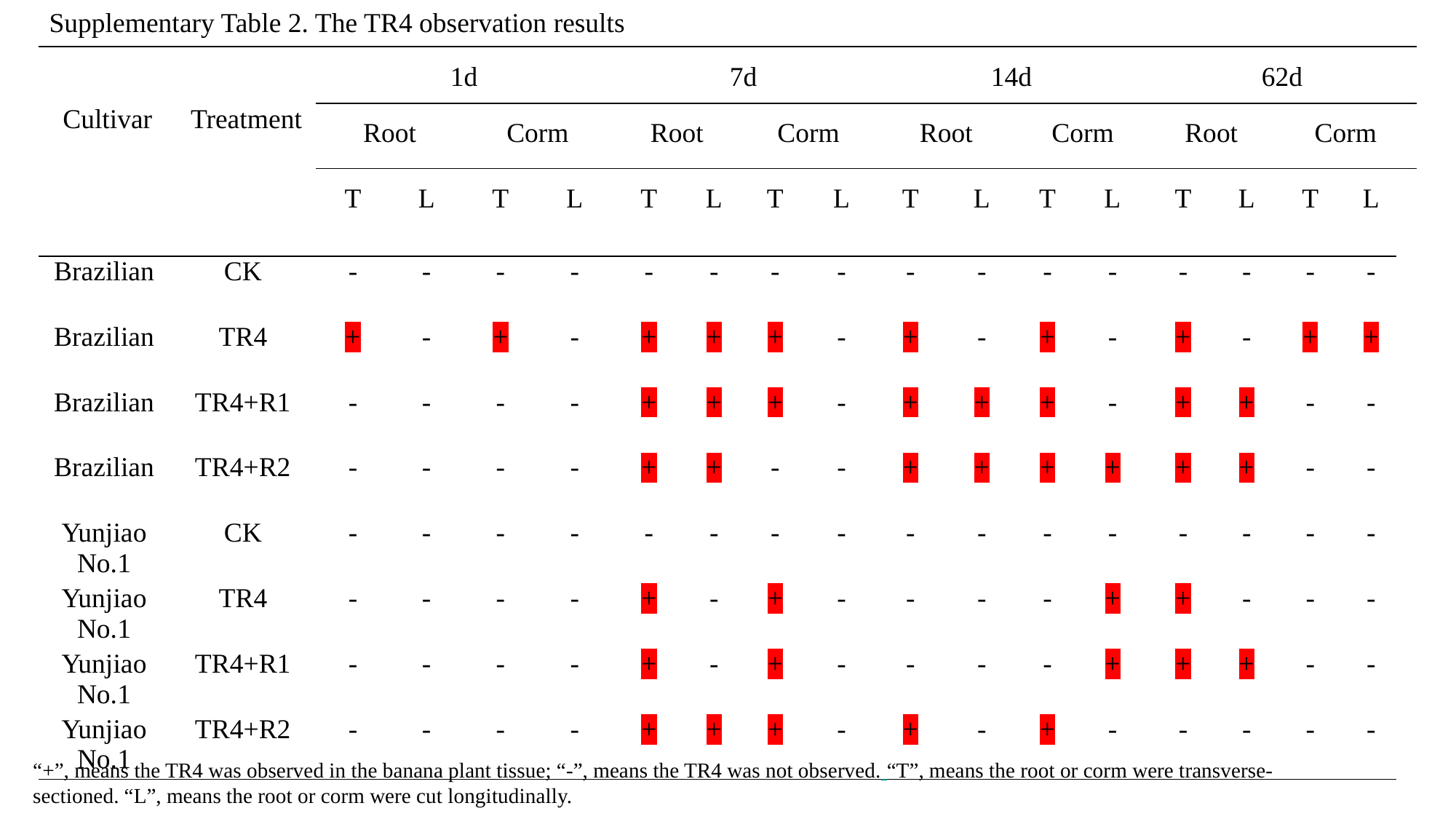

Supplementary Table 2. The TR4 observation results
| Cultivar | Treatment | 1d | | | | 7d | | | | 14d | | | | 62d | | | | |
| --- | --- | --- | --- | --- | --- | --- | --- | --- | --- | --- | --- | --- | --- | --- | --- | --- | --- | --- |
| | | Root | | Corm | | Root | | Corm | | Root | | Corm | | Root | | Corm | | |
| | | T | L | T | L | T | L | T | L | T | L | T | L | T | L | T | L | |
| Brazilian | CK | - | - | - | - | - | - | - | - | - | - | - | - | - | - | - | - | |
| Brazilian | TR4 | + | - | + | - | + | + | + | - | + | - | + | - | + | - | + | + | |
| Brazilian | TR4+R1 | - | - | - | - | + | + | + | - | + | + | + | - | + | + | - | - | |
| Brazilian | TR4+R2 | - | - | - | - | + | + | - | - | + | + | + | + | + | + | - | - | |
| Yunjiao No.1 | CK | - | - | - | - | - | - | - | - | - | - | - | - | - | - | - | - | |
| Yunjiao No.1 | TR4 | - | - | - | - | + | - | + | - | - | - | - | + | + | - | - | - | |
| Yunjiao No.1 | TR4+R1 | - | - | - | - | + | - | + | - | - | - | - | + | + | + | - | - | |
| Yunjiao No.1 | TR4+R2 | - | - | - | - | + | + | + | - | + | - | + | - | - | - | - | - | |
“+”, means the TR4 was observed in the banana plant tissue; “-”, means the TR4 was not observed. “T”, means the root or corm were transverse-sectioned. “L”, means the root or corm were cut longitudinally.

## Slide 3
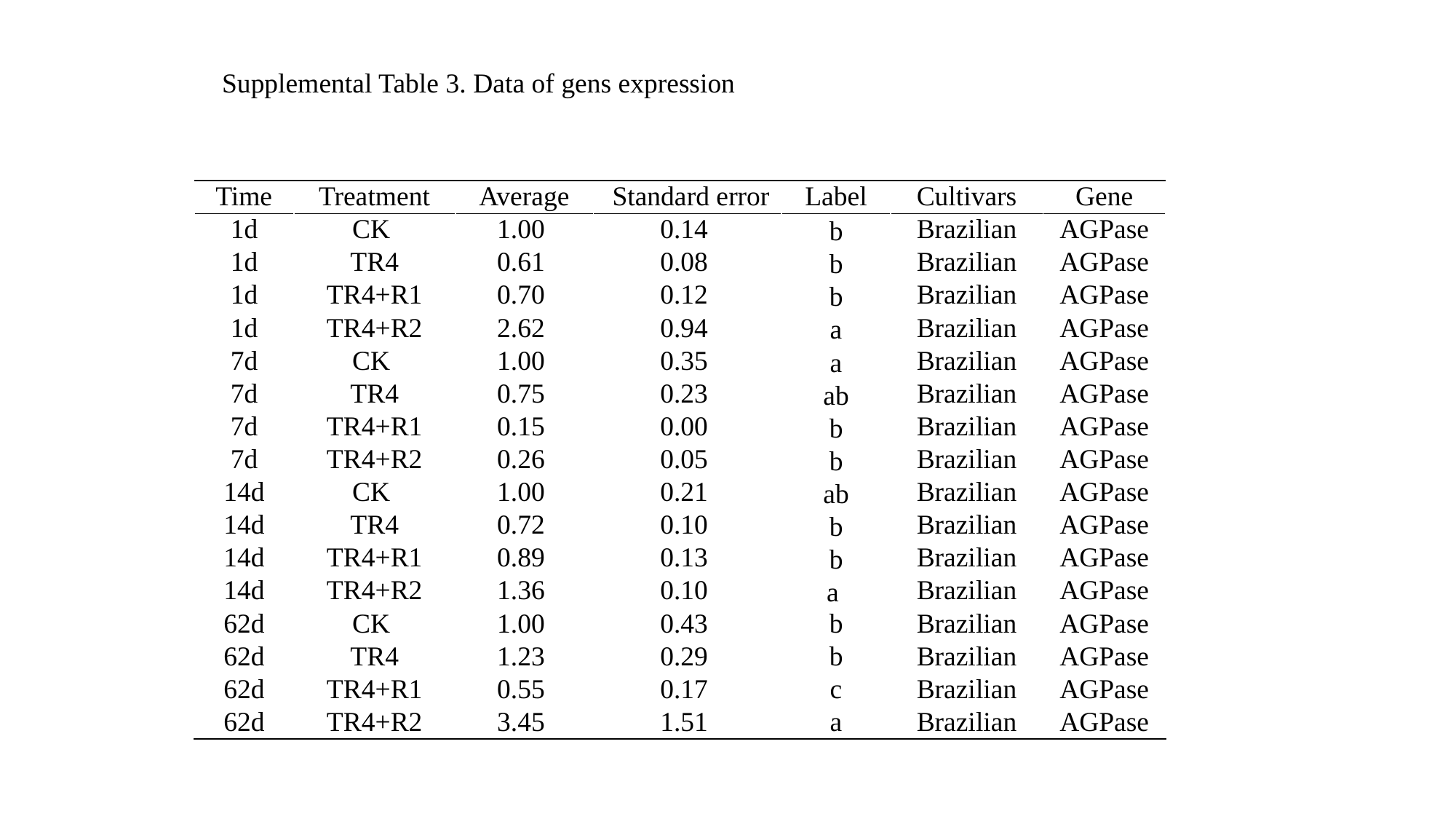

# Supplemental Table 3. Data of gens expression
| Time | Treatment | Average | Standard error | Label | Cultivars | Gene |
| --- | --- | --- | --- | --- | --- | --- |
| 1d | CK | 1.00 | 0.14 | b | Brazilian | AGPase |
| 1d | TR4 | 0.61 | 0.08 | b | Brazilian | AGPase |
| 1d | TR4+R1 | 0.70 | 0.12 | b | Brazilian | AGPase |
| 1d | TR4+R2 | 2.62 | 0.94 | a | Brazilian | AGPase |
| 7d | CK | 1.00 | 0.35 | a | Brazilian | AGPase |
| 7d | TR4 | 0.75 | 0.23 | ab | Brazilian | AGPase |
| 7d | TR4+R1 | 0.15 | 0.00 | b | Brazilian | AGPase |
| 7d | TR4+R2 | 0.26 | 0.05 | b | Brazilian | AGPase |
| 14d | CK | 1.00 | 0.21 | ab | Brazilian | AGPase |
| 14d | TR4 | 0.72 | 0.10 | b | Brazilian | AGPase |
| 14d | TR4+R1 | 0.89 | 0.13 | b | Brazilian | AGPase |
| 14d | TR4+R2 | 1.36 | 0.10 | a | Brazilian | AGPase |
| 62d | CK | 1.00 | 0.43 | b | Brazilian | AGPase |
| 62d | TR4 | 1.23 | 0.29 | b | Brazilian | AGPase |
| 62d | TR4+R1 | 0.55 | 0.17 | c | Brazilian | AGPase |
| 62d | TR4+R2 | 3.45 | 1.51 | a | Brazilian | AGPase |

## Slide 4
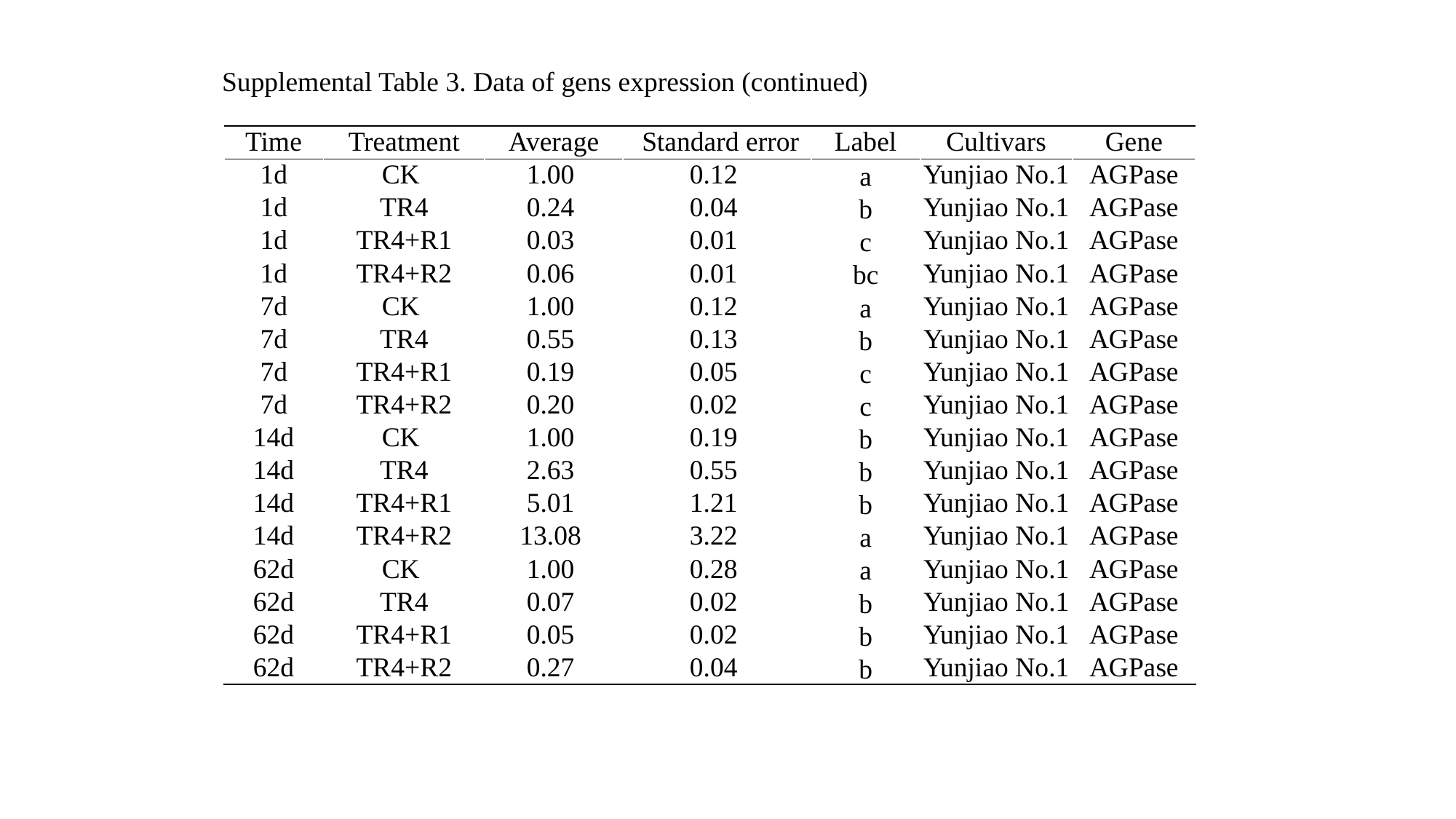

# Supplemental Table 3. Data of gens expression (continued)
| Time | Treatment | Average | Standard error | Label | Cultivars | Gene |
| --- | --- | --- | --- | --- | --- | --- |
| 1d | CK | 1.00 | 0.12 | a | Yunjiao No.1 | AGPase |
| 1d | TR4 | 0.24 | 0.04 | b | Yunjiao No.1 | AGPase |
| 1d | TR4+R1 | 0.03 | 0.01 | c | Yunjiao No.1 | AGPase |
| 1d | TR4+R2 | 0.06 | 0.01 | bc | Yunjiao No.1 | AGPase |
| 7d | CK | 1.00 | 0.12 | a | Yunjiao No.1 | AGPase |
| 7d | TR4 | 0.55 | 0.13 | b | Yunjiao No.1 | AGPase |
| 7d | TR4+R1 | 0.19 | 0.05 | c | Yunjiao No.1 | AGPase |
| 7d | TR4+R2 | 0.20 | 0.02 | c | Yunjiao No.1 | AGPase |
| 14d | CK | 1.00 | 0.19 | b | Yunjiao No.1 | AGPase |
| 14d | TR4 | 2.63 | 0.55 | b | Yunjiao No.1 | AGPase |
| 14d | TR4+R1 | 5.01 | 1.21 | b | Yunjiao No.1 | AGPase |
| 14d | TR4+R2 | 13.08 | 3.22 | a | Yunjiao No.1 | AGPase |
| 62d | CK | 1.00 | 0.28 | a | Yunjiao No.1 | AGPase |
| 62d | TR4 | 0.07 | 0.02 | b | Yunjiao No.1 | AGPase |
| 62d | TR4+R1 | 0.05 | 0.02 | b | Yunjiao No.1 | AGPase |
| 62d | TR4+R2 | 0.27 | 0.04 | b | Yunjiao No.1 | AGPase |

## Slide 5
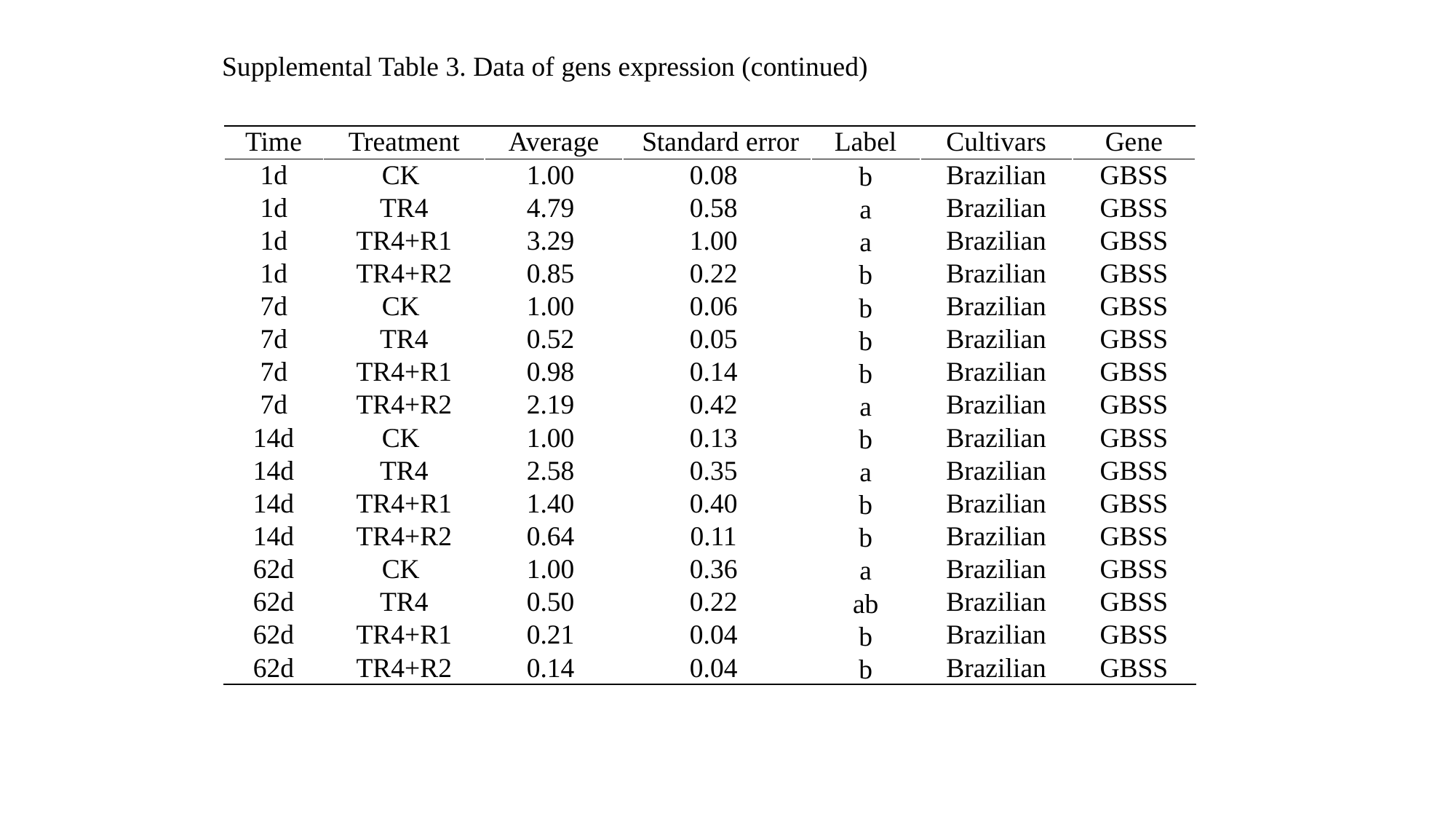

# Supplemental Table 3. Data of gens expression (continued)
| Time | Treatment | Average | Standard error | Label | Cultivars | Gene |
| --- | --- | --- | --- | --- | --- | --- |
| 1d | CK | 1.00 | 0.08 | b | Brazilian | GBSS |
| 1d | TR4 | 4.79 | 0.58 | a | Brazilian | GBSS |
| 1d | TR4+R1 | 3.29 | 1.00 | a | Brazilian | GBSS |
| 1d | TR4+R2 | 0.85 | 0.22 | b | Brazilian | GBSS |
| 7d | CK | 1.00 | 0.06 | b | Brazilian | GBSS |
| 7d | TR4 | 0.52 | 0.05 | b | Brazilian | GBSS |
| 7d | TR4+R1 | 0.98 | 0.14 | b | Brazilian | GBSS |
| 7d | TR4+R2 | 2.19 | 0.42 | a | Brazilian | GBSS |
| 14d | CK | 1.00 | 0.13 | b | Brazilian | GBSS |
| 14d | TR4 | 2.58 | 0.35 | a | Brazilian | GBSS |
| 14d | TR4+R1 | 1.40 | 0.40 | b | Brazilian | GBSS |
| 14d | TR4+R2 | 0.64 | 0.11 | b | Brazilian | GBSS |
| 62d | CK | 1.00 | 0.36 | a | Brazilian | GBSS |
| 62d | TR4 | 0.50 | 0.22 | ab | Brazilian | GBSS |
| 62d | TR4+R1 | 0.21 | 0.04 | b | Brazilian | GBSS |
| 62d | TR4+R2 | 0.14 | 0.04 | b | Brazilian | GBSS |

## Slide 6
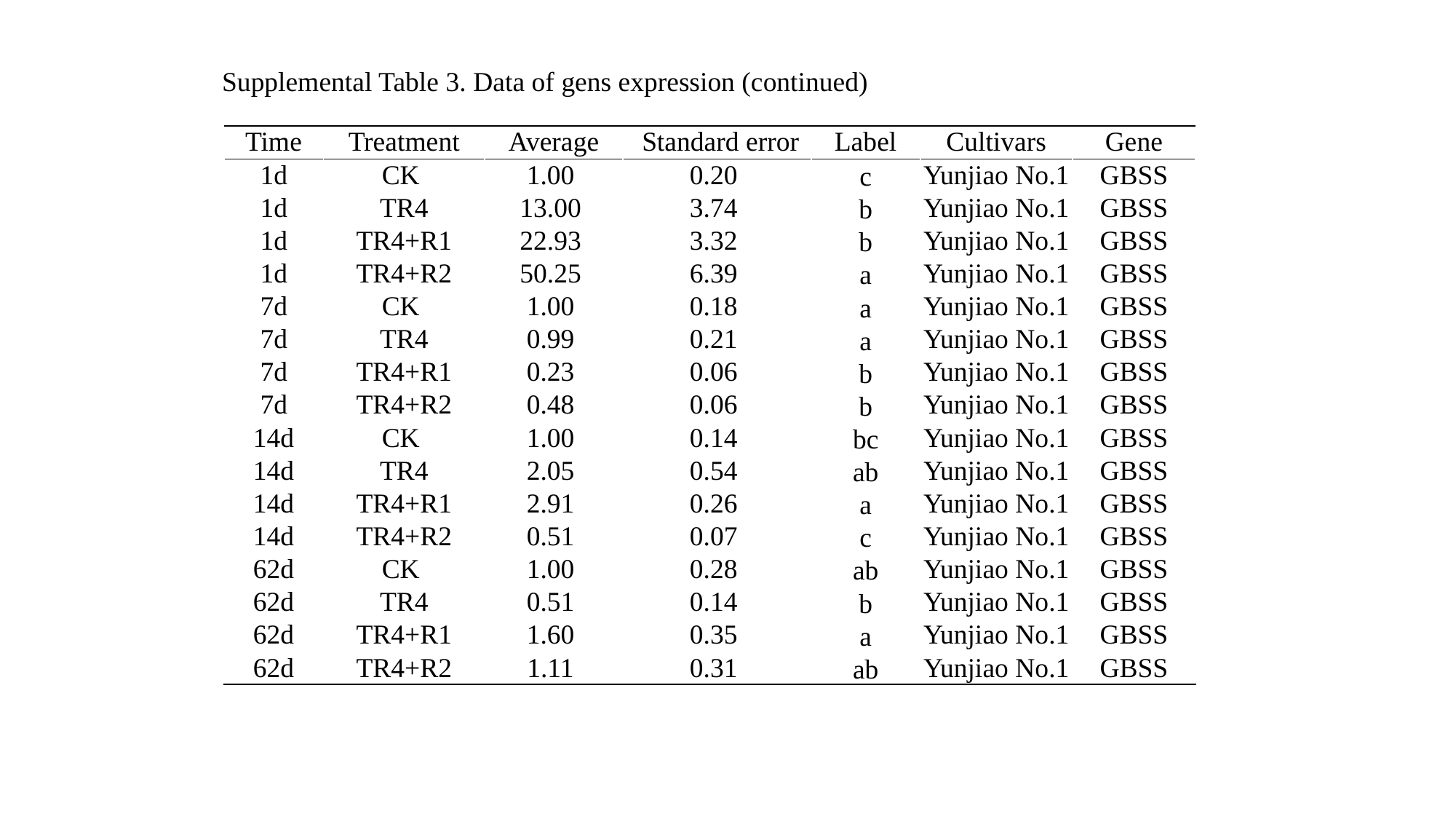

# Supplemental Table 3. Data of gens expression (continued)
| Time | Treatment | Average | Standard error | Label | Cultivars | Gene |
| --- | --- | --- | --- | --- | --- | --- |
| 1d | CK | 1.00 | 0.20 | c | Yunjiao No.1 | GBSS |
| 1d | TR4 | 13.00 | 3.74 | b | Yunjiao No.1 | GBSS |
| 1d | TR4+R1 | 22.93 | 3.32 | b | Yunjiao No.1 | GBSS |
| 1d | TR4+R2 | 50.25 | 6.39 | a | Yunjiao No.1 | GBSS |
| 7d | CK | 1.00 | 0.18 | a | Yunjiao No.1 | GBSS |
| 7d | TR4 | 0.99 | 0.21 | a | Yunjiao No.1 | GBSS |
| 7d | TR4+R1 | 0.23 | 0.06 | b | Yunjiao No.1 | GBSS |
| 7d | TR4+R2 | 0.48 | 0.06 | b | Yunjiao No.1 | GBSS |
| 14d | CK | 1.00 | 0.14 | bc | Yunjiao No.1 | GBSS |
| 14d | TR4 | 2.05 | 0.54 | ab | Yunjiao No.1 | GBSS |
| 14d | TR4+R1 | 2.91 | 0.26 | a | Yunjiao No.1 | GBSS |
| 14d | TR4+R2 | 0.51 | 0.07 | c | Yunjiao No.1 | GBSS |
| 62d | CK | 1.00 | 0.28 | ab | Yunjiao No.1 | GBSS |
| 62d | TR4 | 0.51 | 0.14 | b | Yunjiao No.1 | GBSS |
| 62d | TR4+R1 | 1.60 | 0.35 | a | Yunjiao No.1 | GBSS |
| 62d | TR4+R2 | 1.11 | 0.31 | ab | Yunjiao No.1 | GBSS |

## Slide 7
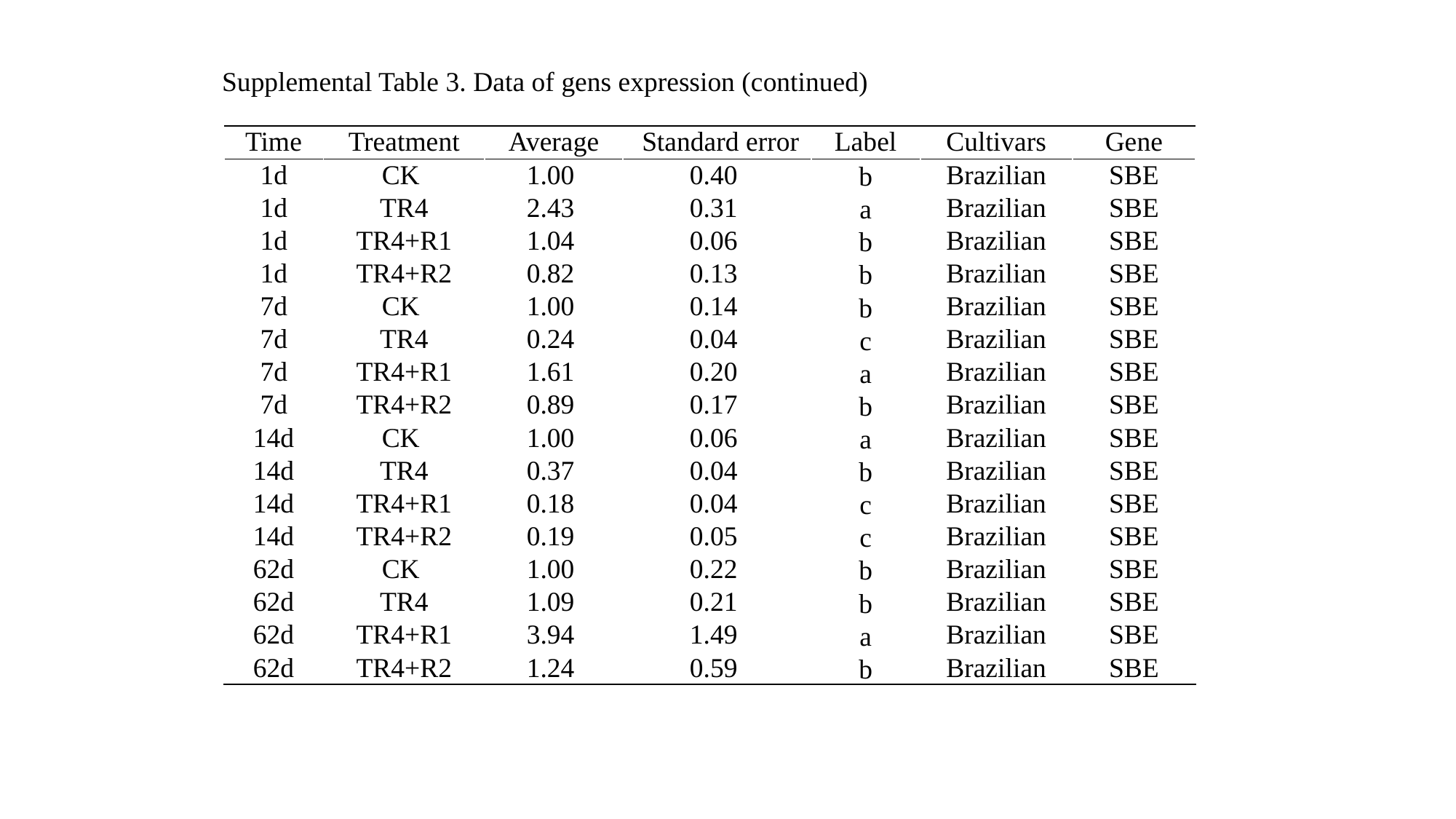

# Supplemental Table 3. Data of gens expression (continued)
| Time | Treatment | Average | Standard error | Label | Cultivars | Gene |
| --- | --- | --- | --- | --- | --- | --- |
| 1d | CK | 1.00 | 0.40 | b | Brazilian | SBE |
| 1d | TR4 | 2.43 | 0.31 | a | Brazilian | SBE |
| 1d | TR4+R1 | 1.04 | 0.06 | b | Brazilian | SBE |
| 1d | TR4+R2 | 0.82 | 0.13 | b | Brazilian | SBE |
| 7d | CK | 1.00 | 0.14 | b | Brazilian | SBE |
| 7d | TR4 | 0.24 | 0.04 | c | Brazilian | SBE |
| 7d | TR4+R1 | 1.61 | 0.20 | a | Brazilian | SBE |
| 7d | TR4+R2 | 0.89 | 0.17 | b | Brazilian | SBE |
| 14d | CK | 1.00 | 0.06 | a | Brazilian | SBE |
| 14d | TR4 | 0.37 | 0.04 | b | Brazilian | SBE |
| 14d | TR4+R1 | 0.18 | 0.04 | c | Brazilian | SBE |
| 14d | TR4+R2 | 0.19 | 0.05 | c | Brazilian | SBE |
| 62d | CK | 1.00 | 0.22 | b | Brazilian | SBE |
| 62d | TR4 | 1.09 | 0.21 | b | Brazilian | SBE |
| 62d | TR4+R1 | 3.94 | 1.49 | a | Brazilian | SBE |
| 62d | TR4+R2 | 1.24 | 0.59 | b | Brazilian | SBE |

## Slide 8
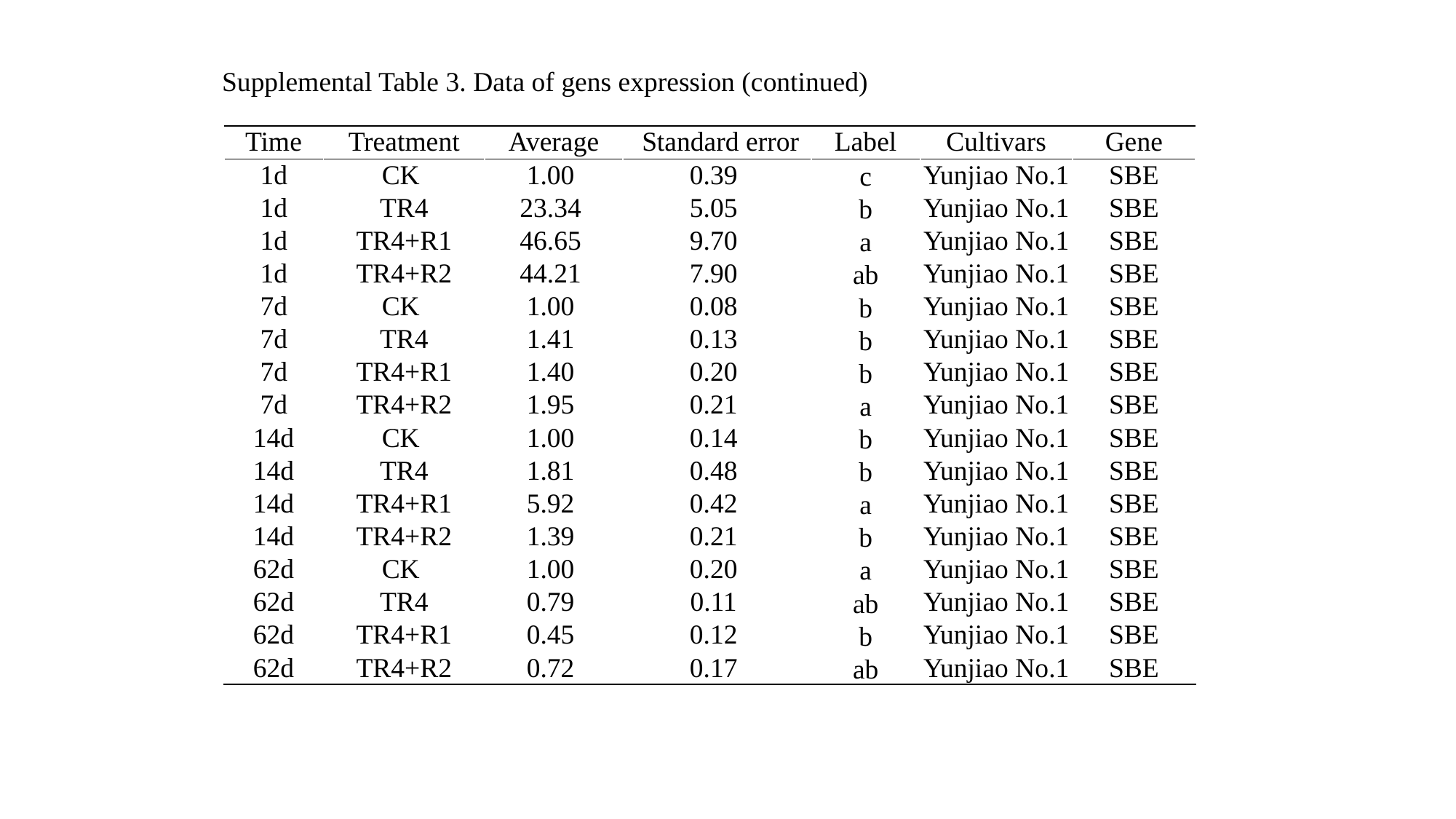

# Supplemental Table 3. Data of gens expression (continued)
| Time | Treatment | Average | Standard error | Label | Cultivars | Gene |
| --- | --- | --- | --- | --- | --- | --- |
| 1d | CK | 1.00 | 0.39 | c | Yunjiao No.1 | SBE |
| 1d | TR4 | 23.34 | 5.05 | b | Yunjiao No.1 | SBE |
| 1d | TR4+R1 | 46.65 | 9.70 | a | Yunjiao No.1 | SBE |
| 1d | TR4+R2 | 44.21 | 7.90 | ab | Yunjiao No.1 | SBE |
| 7d | CK | 1.00 | 0.08 | b | Yunjiao No.1 | SBE |
| 7d | TR4 | 1.41 | 0.13 | b | Yunjiao No.1 | SBE |
| 7d | TR4+R1 | 1.40 | 0.20 | b | Yunjiao No.1 | SBE |
| 7d | TR4+R2 | 1.95 | 0.21 | a | Yunjiao No.1 | SBE |
| 14d | CK | 1.00 | 0.14 | b | Yunjiao No.1 | SBE |
| 14d | TR4 | 1.81 | 0.48 | b | Yunjiao No.1 | SBE |
| 14d | TR4+R1 | 5.92 | 0.42 | a | Yunjiao No.1 | SBE |
| 14d | TR4+R2 | 1.39 | 0.21 | b | Yunjiao No.1 | SBE |
| 62d | CK | 1.00 | 0.20 | a | Yunjiao No.1 | SBE |
| 62d | TR4 | 0.79 | 0.11 | ab | Yunjiao No.1 | SBE |
| 62d | TR4+R1 | 0.45 | 0.12 | b | Yunjiao No.1 | SBE |
| 62d | TR4+R2 | 0.72 | 0.17 | ab | Yunjiao No.1 | SBE |

## Slide 9
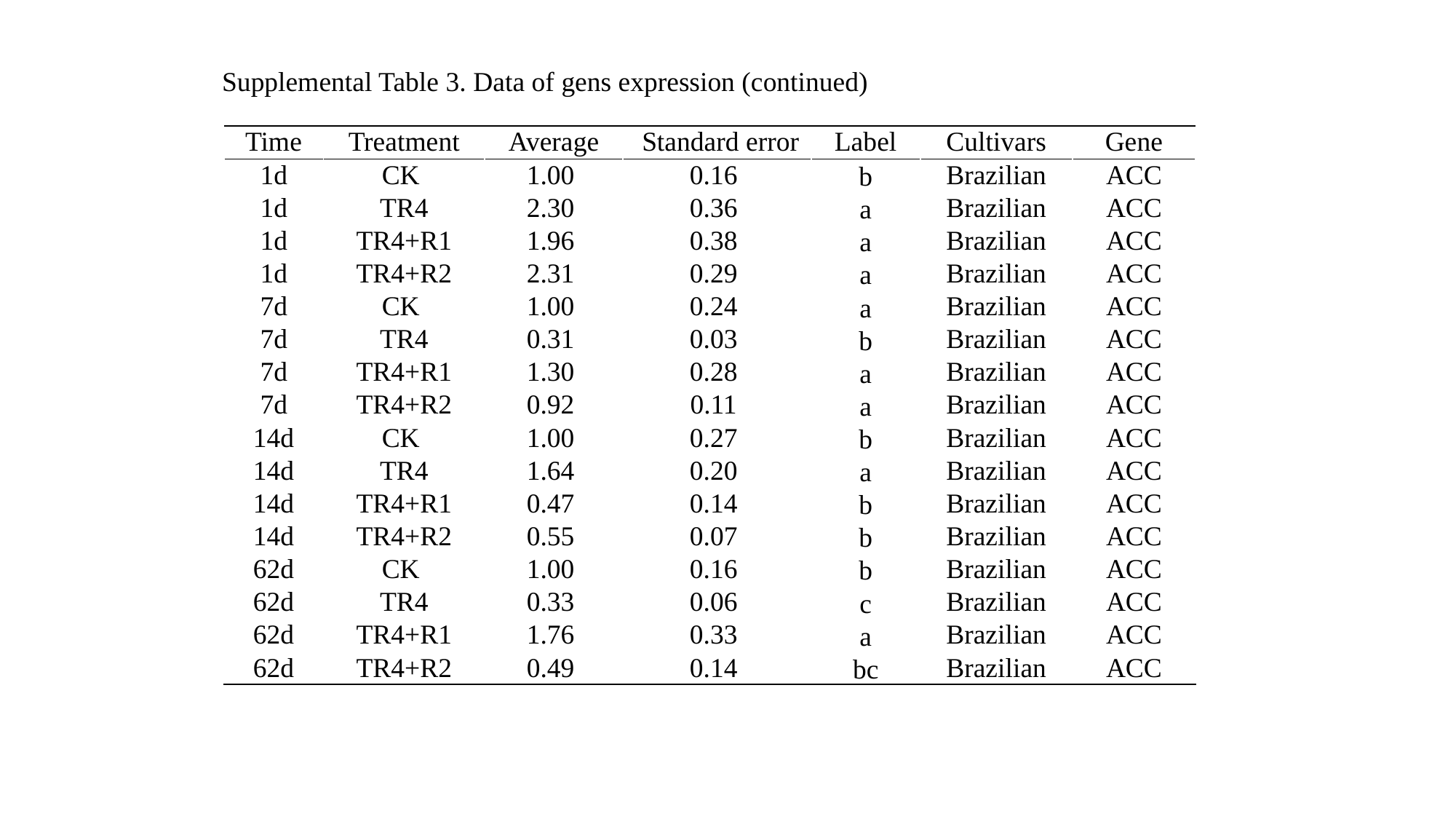

# Supplemental Table 3. Data of gens expression (continued)
| Time | Treatment | Average | Standard error | Label | Cultivars | Gene |
| --- | --- | --- | --- | --- | --- | --- |
| 1d | CK | 1.00 | 0.16 | b | Brazilian | ACC |
| 1d | TR4 | 2.30 | 0.36 | a | Brazilian | ACC |
| 1d | TR4+R1 | 1.96 | 0.38 | a | Brazilian | ACC |
| 1d | TR4+R2 | 2.31 | 0.29 | a | Brazilian | ACC |
| 7d | CK | 1.00 | 0.24 | a | Brazilian | ACC |
| 7d | TR4 | 0.31 | 0.03 | b | Brazilian | ACC |
| 7d | TR4+R1 | 1.30 | 0.28 | a | Brazilian | ACC |
| 7d | TR4+R2 | 0.92 | 0.11 | a | Brazilian | ACC |
| 14d | CK | 1.00 | 0.27 | b | Brazilian | ACC |
| 14d | TR4 | 1.64 | 0.20 | a | Brazilian | ACC |
| 14d | TR4+R1 | 0.47 | 0.14 | b | Brazilian | ACC |
| 14d | TR4+R2 | 0.55 | 0.07 | b | Brazilian | ACC |
| 62d | CK | 1.00 | 0.16 | b | Brazilian | ACC |
| 62d | TR4 | 0.33 | 0.06 | c | Brazilian | ACC |
| 62d | TR4+R1 | 1.76 | 0.33 | a | Brazilian | ACC |
| 62d | TR4+R2 | 0.49 | 0.14 | bc | Brazilian | ACC |

## Slide 10
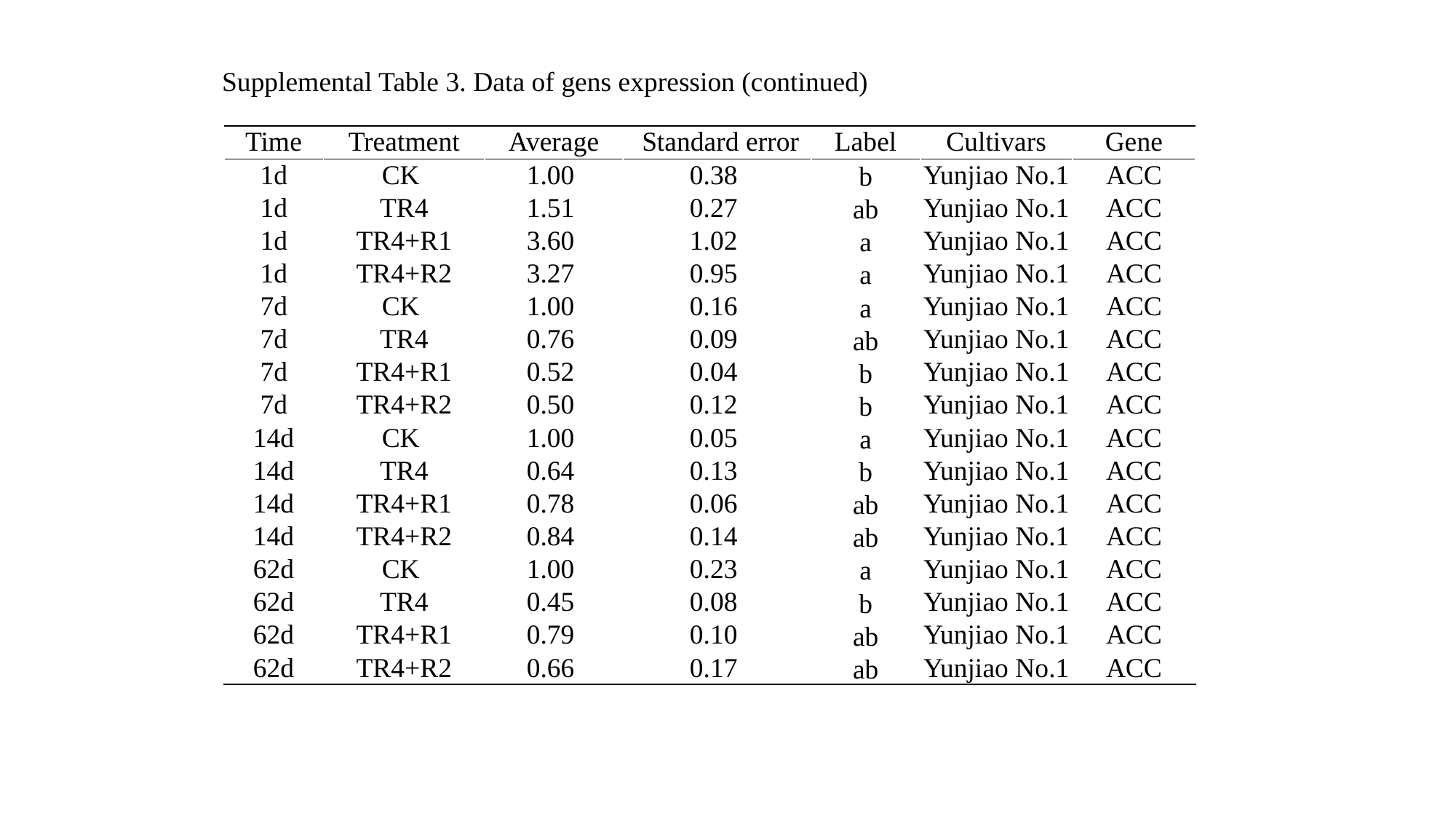

# Supplemental Table 3. Data of gens expression (continued)
| Time | Treatment | Average | Standard error | Label | Cultivars | Gene |
| --- | --- | --- | --- | --- | --- | --- |
| 1d | CK | 1.00 | 0.38 | b | Yunjiao No.1 | ACC |
| 1d | TR4 | 1.51 | 0.27 | ab | Yunjiao No.1 | ACC |
| 1d | TR4+R1 | 3.60 | 1.02 | a | Yunjiao No.1 | ACC |
| 1d | TR4+R2 | 3.27 | 0.95 | a | Yunjiao No.1 | ACC |
| 7d | CK | 1.00 | 0.16 | a | Yunjiao No.1 | ACC |
| 7d | TR4 | 0.76 | 0.09 | ab | Yunjiao No.1 | ACC |
| 7d | TR4+R1 | 0.52 | 0.04 | b | Yunjiao No.1 | ACC |
| 7d | TR4+R2 | 0.50 | 0.12 | b | Yunjiao No.1 | ACC |
| 14d | CK | 1.00 | 0.05 | a | Yunjiao No.1 | ACC |
| 14d | TR4 | 0.64 | 0.13 | b | Yunjiao No.1 | ACC |
| 14d | TR4+R1 | 0.78 | 0.06 | ab | Yunjiao No.1 | ACC |
| 14d | TR4+R2 | 0.84 | 0.14 | ab | Yunjiao No.1 | ACC |
| 62d | CK | 1.00 | 0.23 | a | Yunjiao No.1 | ACC |
| 62d | TR4 | 0.45 | 0.08 | b | Yunjiao No.1 | ACC |
| 62d | TR4+R1 | 0.79 | 0.10 | ab | Yunjiao No.1 | ACC |
| 62d | TR4+R2 | 0.66 | 0.17 | ab | Yunjiao No.1 | ACC |

## Slide 11
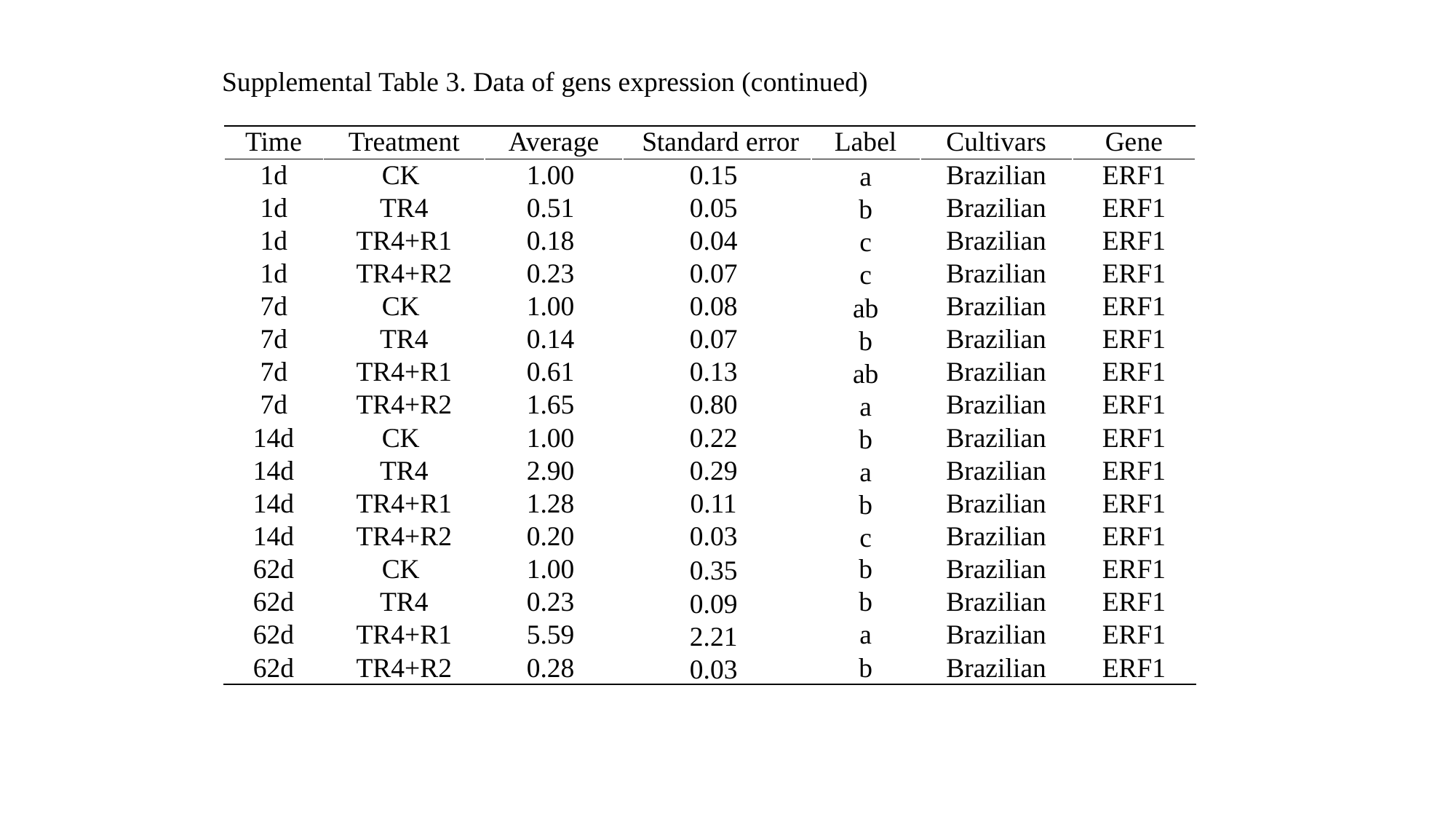

# Supplemental Table 3. Data of gens expression (continued)
| Time | Treatment | Average | Standard error | Label | Cultivars | Gene |
| --- | --- | --- | --- | --- | --- | --- |
| 1d | CK | 1.00 | 0.15 | a | Brazilian | ERF1 |
| 1d | TR4 | 0.51 | 0.05 | b | Brazilian | ERF1 |
| 1d | TR4+R1 | 0.18 | 0.04 | c | Brazilian | ERF1 |
| 1d | TR4+R2 | 0.23 | 0.07 | c | Brazilian | ERF1 |
| 7d | CK | 1.00 | 0.08 | ab | Brazilian | ERF1 |
| 7d | TR4 | 0.14 | 0.07 | b | Brazilian | ERF1 |
| 7d | TR4+R1 | 0.61 | 0.13 | ab | Brazilian | ERF1 |
| 7d | TR4+R2 | 1.65 | 0.80 | a | Brazilian | ERF1 |
| 14d | CK | 1.00 | 0.22 | b | Brazilian | ERF1 |
| 14d | TR4 | 2.90 | 0.29 | a | Brazilian | ERF1 |
| 14d | TR4+R1 | 1.28 | 0.11 | b | Brazilian | ERF1 |
| 14d | TR4+R2 | 0.20 | 0.03 | c | Brazilian | ERF1 |
| 62d | CK | 1.00 | 0.35 | b | Brazilian | ERF1 |
| 62d | TR4 | 0.23 | 0.09 | b | Brazilian | ERF1 |
| 62d | TR4+R1 | 5.59 | 2.21 | a | Brazilian | ERF1 |
| 62d | TR4+R2 | 0.28 | 0.03 | b | Brazilian | ERF1 |

## Slide 12
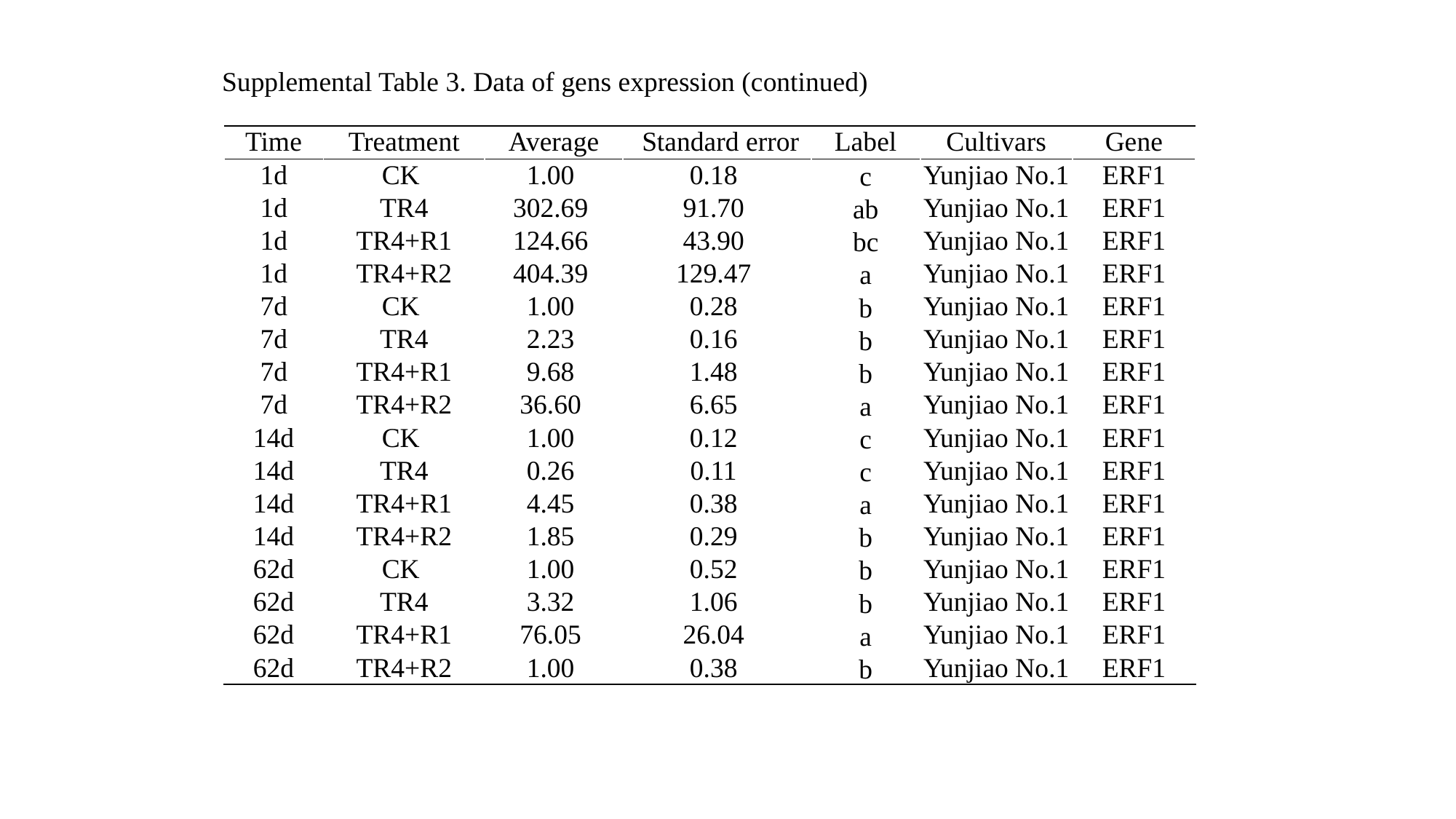

# Supplemental Table 3. Data of gens expression (continued)
| Time | Treatment | Average | Standard error | Label | Cultivars | Gene |
| --- | --- | --- | --- | --- | --- | --- |
| 1d | CK | 1.00 | 0.18 | c | Yunjiao No.1 | ERF1 |
| 1d | TR4 | 302.69 | 91.70 | ab | Yunjiao No.1 | ERF1 |
| 1d | TR4+R1 | 124.66 | 43.90 | bc | Yunjiao No.1 | ERF1 |
| 1d | TR4+R2 | 404.39 | 129.47 | a | Yunjiao No.1 | ERF1 |
| 7d | CK | 1.00 | 0.28 | b | Yunjiao No.1 | ERF1 |
| 7d | TR4 | 2.23 | 0.16 | b | Yunjiao No.1 | ERF1 |
| 7d | TR4+R1 | 9.68 | 1.48 | b | Yunjiao No.1 | ERF1 |
| 7d | TR4+R2 | 36.60 | 6.65 | a | Yunjiao No.1 | ERF1 |
| 14d | CK | 1.00 | 0.12 | c | Yunjiao No.1 | ERF1 |
| 14d | TR4 | 0.26 | 0.11 | c | Yunjiao No.1 | ERF1 |
| 14d | TR4+R1 | 4.45 | 0.38 | a | Yunjiao No.1 | ERF1 |
| 14d | TR4+R2 | 1.85 | 0.29 | b | Yunjiao No.1 | ERF1 |
| 62d | CK | 1.00 | 0.52 | b | Yunjiao No.1 | ERF1 |
| 62d | TR4 | 3.32 | 1.06 | b | Yunjiao No.1 | ERF1 |
| 62d | TR4+R1 | 76.05 | 26.04 | a | Yunjiao No.1 | ERF1 |
| 62d | TR4+R2 | 1.00 | 0.38 | b | Yunjiao No.1 | ERF1 |

## Slide 13
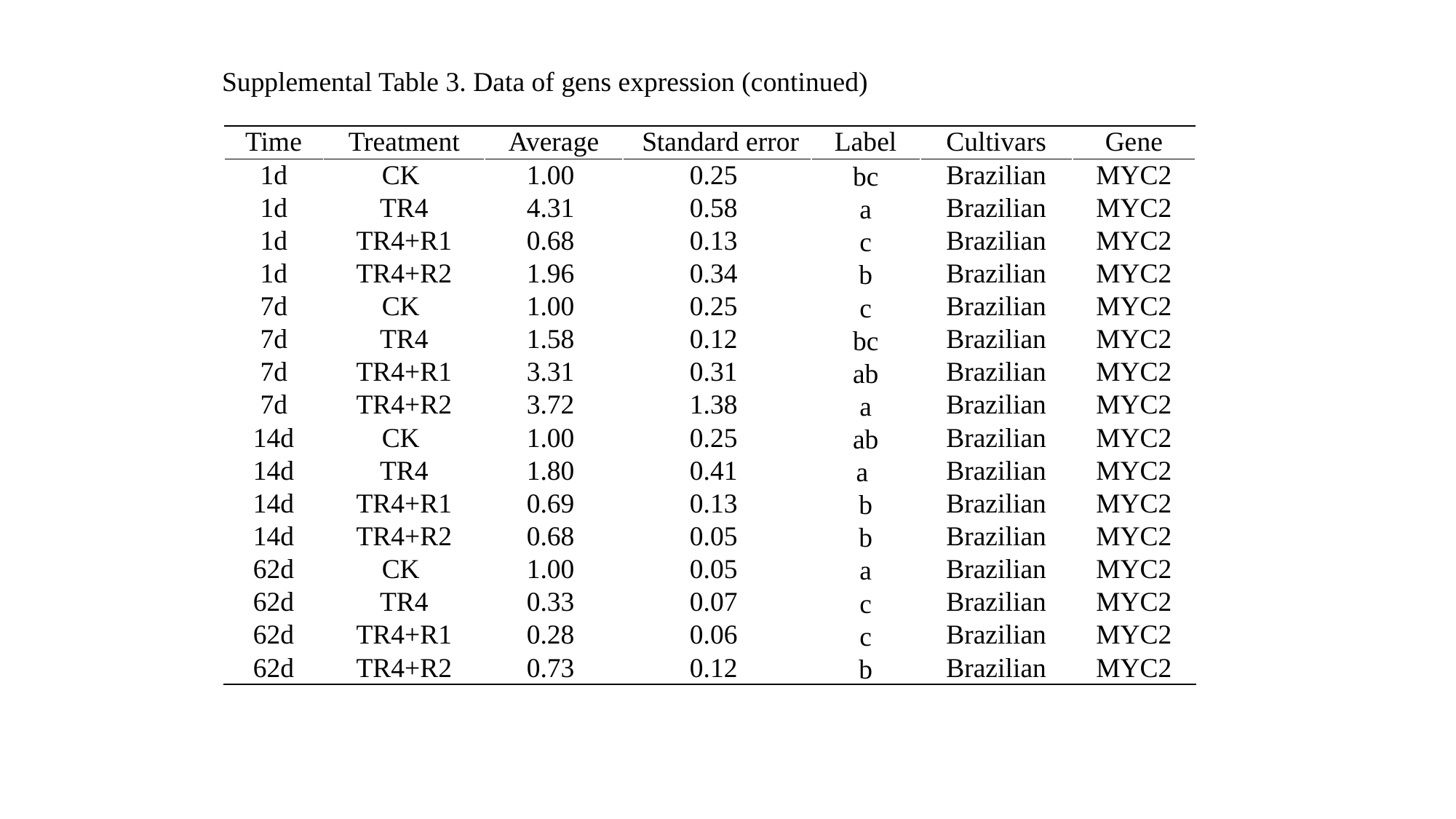

# Supplemental Table 3. Data of gens expression (continued)
| Time | Treatment | Average | Standard error | Label | Cultivars | Gene |
| --- | --- | --- | --- | --- | --- | --- |
| 1d | CK | 1.00 | 0.25 | bc | Brazilian | MYC2 |
| 1d | TR4 | 4.31 | 0.58 | a | Brazilian | MYC2 |
| 1d | TR4+R1 | 0.68 | 0.13 | c | Brazilian | MYC2 |
| 1d | TR4+R2 | 1.96 | 0.34 | b | Brazilian | MYC2 |
| 7d | CK | 1.00 | 0.25 | c | Brazilian | MYC2 |
| 7d | TR4 | 1.58 | 0.12 | bc | Brazilian | MYC2 |
| 7d | TR4+R1 | 3.31 | 0.31 | ab | Brazilian | MYC2 |
| 7d | TR4+R2 | 3.72 | 1.38 | a | Brazilian | MYC2 |
| 14d | CK | 1.00 | 0.25 | ab | Brazilian | MYC2 |
| 14d | TR4 | 1.80 | 0.41 | a | Brazilian | MYC2 |
| 14d | TR4+R1 | 0.69 | 0.13 | b | Brazilian | MYC2 |
| 14d | TR4+R2 | 0.68 | 0.05 | b | Brazilian | MYC2 |
| 62d | CK | 1.00 | 0.05 | a | Brazilian | MYC2 |
| 62d | TR4 | 0.33 | 0.07 | c | Brazilian | MYC2 |
| 62d | TR4+R1 | 0.28 | 0.06 | c | Brazilian | MYC2 |
| 62d | TR4+R2 | 0.73 | 0.12 | b | Brazilian | MYC2 |

## Slide 14
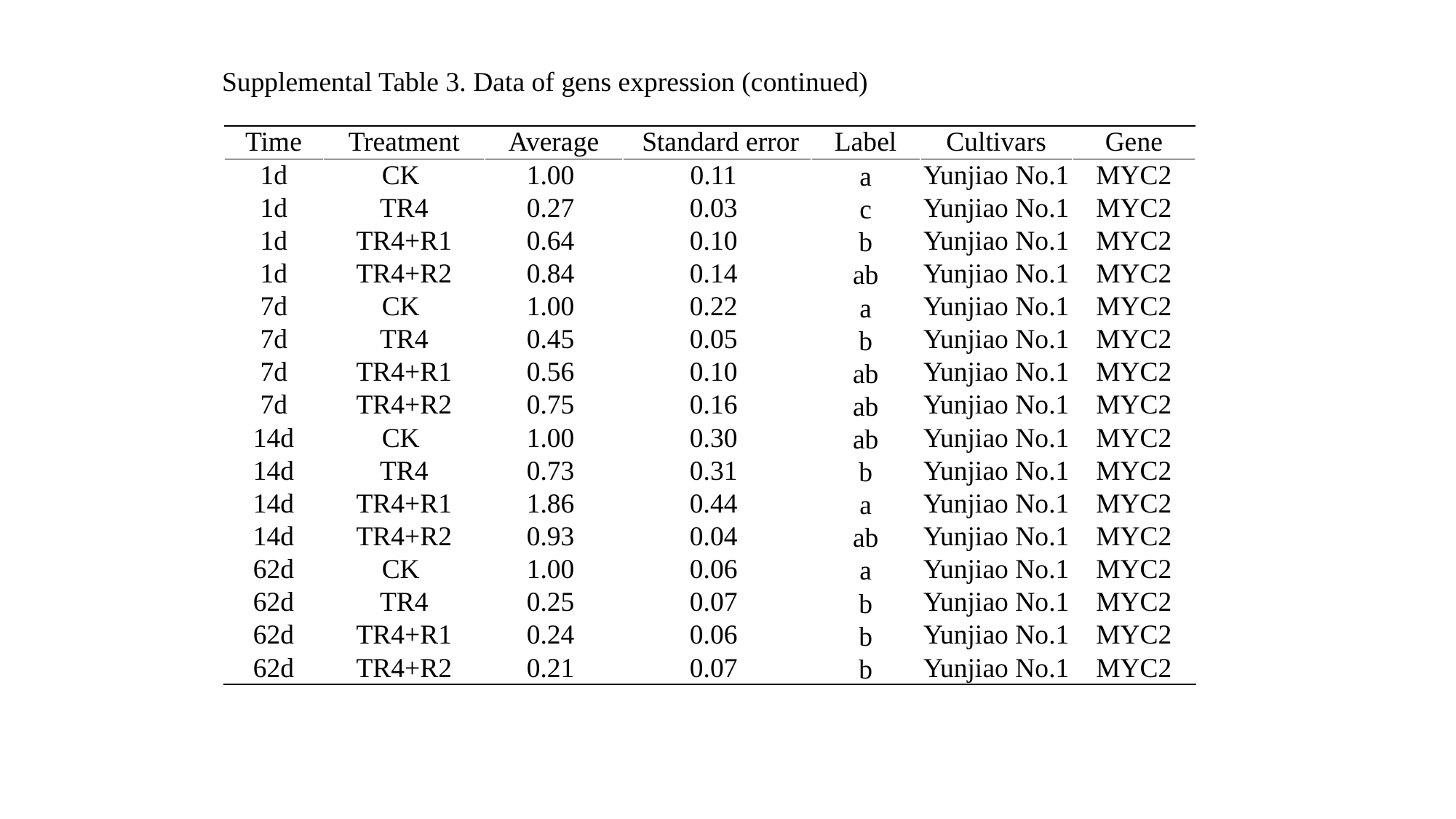

# Supplemental Table 3. Data of gens expression (continued)
| Time | Treatment | Average | Standard error | Label | Cultivars | Gene |
| --- | --- | --- | --- | --- | --- | --- |
| 1d | CK | 1.00 | 0.11 | a | Yunjiao No.1 | MYC2 |
| 1d | TR4 | 0.27 | 0.03 | c | Yunjiao No.1 | MYC2 |
| 1d | TR4+R1 | 0.64 | 0.10 | b | Yunjiao No.1 | MYC2 |
| 1d | TR4+R2 | 0.84 | 0.14 | ab | Yunjiao No.1 | MYC2 |
| 7d | CK | 1.00 | 0.22 | a | Yunjiao No.1 | MYC2 |
| 7d | TR4 | 0.45 | 0.05 | b | Yunjiao No.1 | MYC2 |
| 7d | TR4+R1 | 0.56 | 0.10 | ab | Yunjiao No.1 | MYC2 |
| 7d | TR4+R2 | 0.75 | 0.16 | ab | Yunjiao No.1 | MYC2 |
| 14d | CK | 1.00 | 0.30 | ab | Yunjiao No.1 | MYC2 |
| 14d | TR4 | 0.73 | 0.31 | b | Yunjiao No.1 | MYC2 |
| 14d | TR4+R1 | 1.86 | 0.44 | a | Yunjiao No.1 | MYC2 |
| 14d | TR4+R2 | 0.93 | 0.04 | ab | Yunjiao No.1 | MYC2 |
| 62d | CK | 1.00 | 0.06 | a | Yunjiao No.1 | MYC2 |
| 62d | TR4 | 0.25 | 0.07 | b | Yunjiao No.1 | MYC2 |
| 62d | TR4+R1 | 0.24 | 0.06 | b | Yunjiao No.1 | MYC2 |
| 62d | TR4+R2 | 0.21 | 0.07 | b | Yunjiao No.1 | MYC2 |

## Slide 15
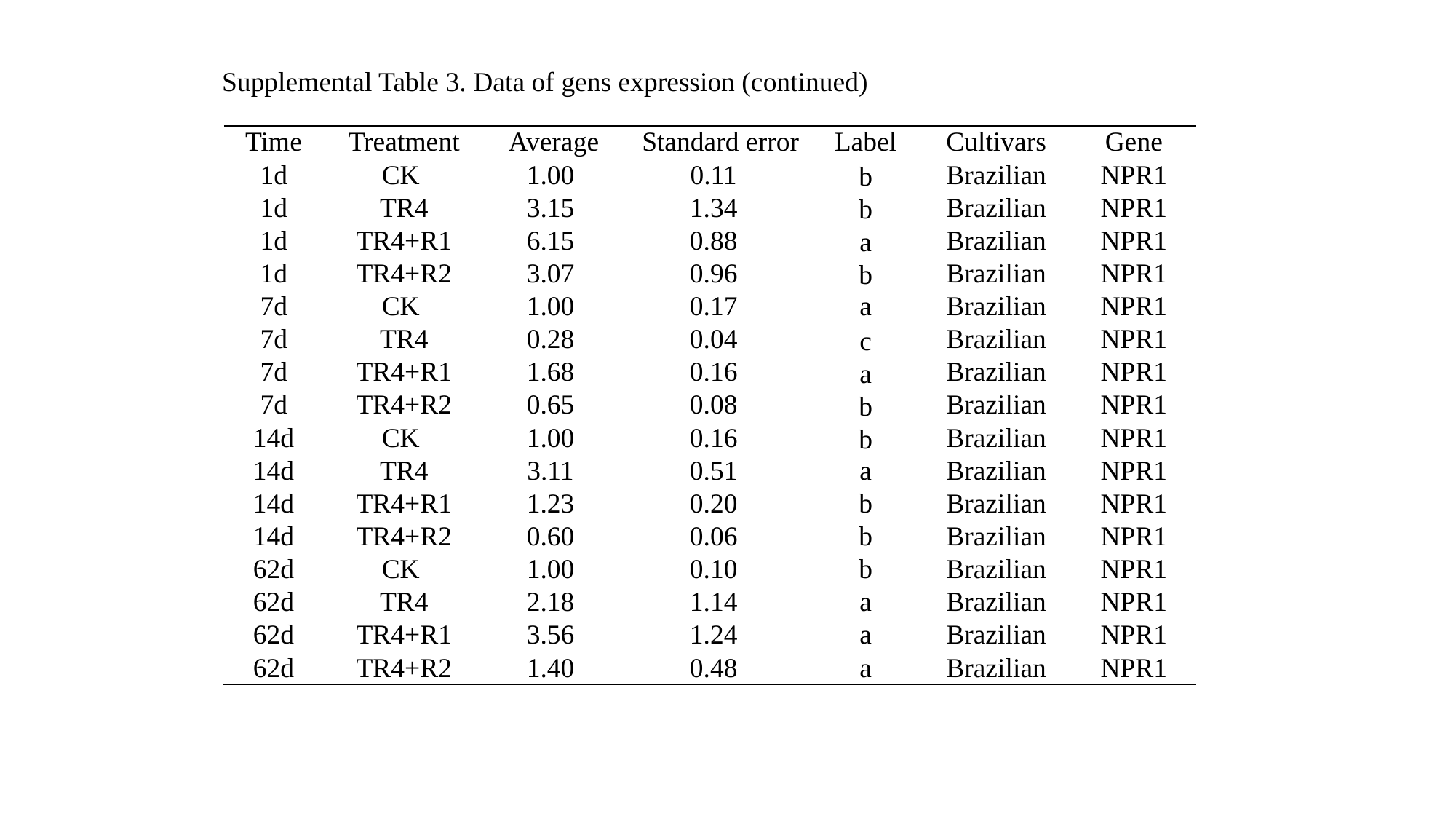

# Supplemental Table 3. Data of gens expression (continued)
| Time | Treatment | Average | Standard error | Label | Cultivars | Gene |
| --- | --- | --- | --- | --- | --- | --- |
| 1d | CK | 1.00 | 0.11 | b | Brazilian | NPR1 |
| 1d | TR4 | 3.15 | 1.34 | b | Brazilian | NPR1 |
| 1d | TR4+R1 | 6.15 | 0.88 | a | Brazilian | NPR1 |
| 1d | TR4+R2 | 3.07 | 0.96 | b | Brazilian | NPR1 |
| 7d | CK | 1.00 | 0.17 | a | Brazilian | NPR1 |
| 7d | TR4 | 0.28 | 0.04 | c | Brazilian | NPR1 |
| 7d | TR4+R1 | 1.68 | 0.16 | a | Brazilian | NPR1 |
| 7d | TR4+R2 | 0.65 | 0.08 | b | Brazilian | NPR1 |
| 14d | CK | 1.00 | 0.16 | b | Brazilian | NPR1 |
| 14d | TR4 | 3.11 | 0.51 | a | Brazilian | NPR1 |
| 14d | TR4+R1 | 1.23 | 0.20 | b | Brazilian | NPR1 |
| 14d | TR4+R2 | 0.60 | 0.06 | b | Brazilian | NPR1 |
| 62d | CK | 1.00 | 0.10 | b | Brazilian | NPR1 |
| 62d | TR4 | 2.18 | 1.14 | a | Brazilian | NPR1 |
| 62d | TR4+R1 | 3.56 | 1.24 | a | Brazilian | NPR1 |
| 62d | TR4+R2 | 1.40 | 0.48 | a | Brazilian | NPR1 |

## Slide 16
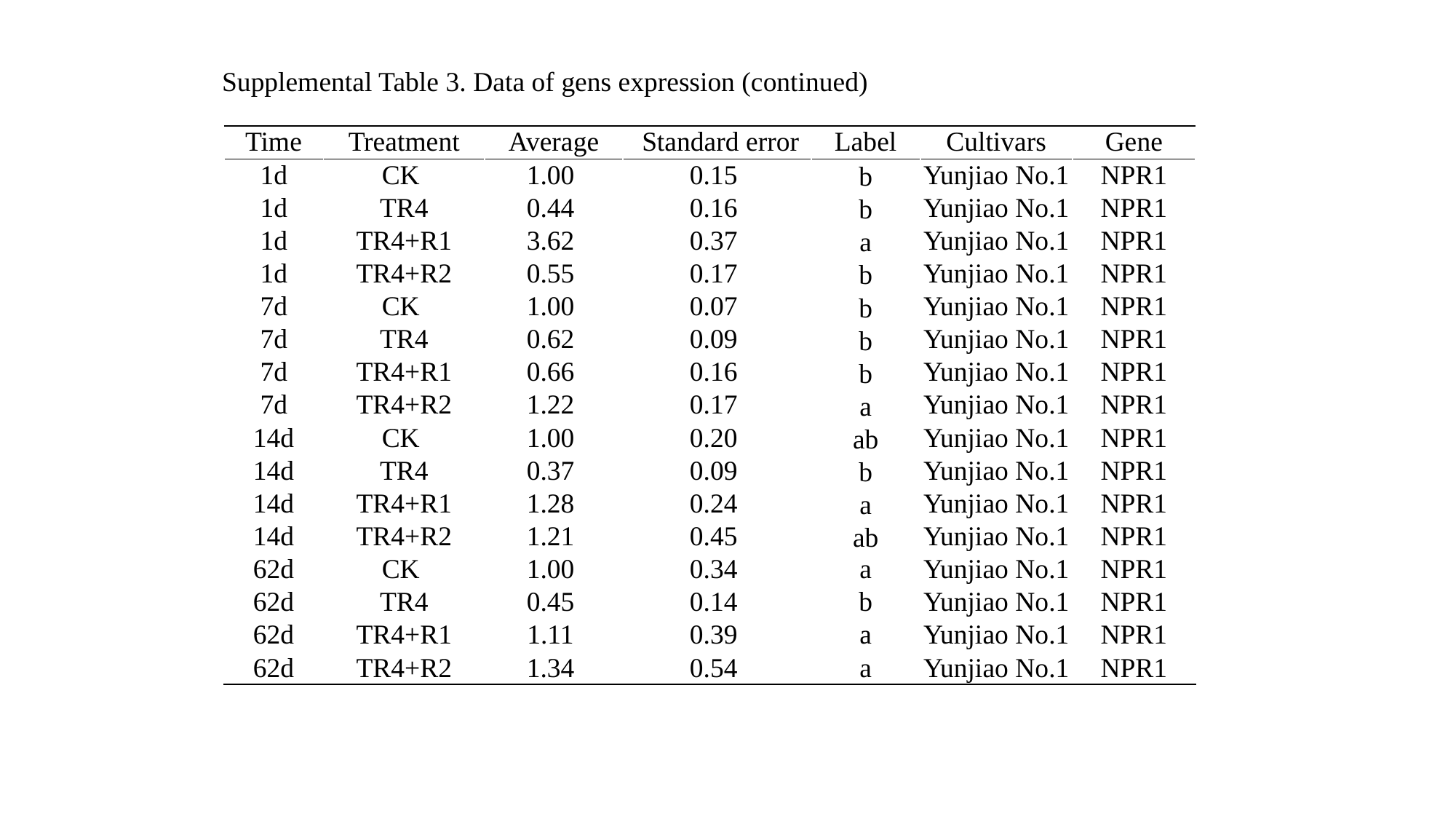

# Supplemental Table 3. Data of gens expression (continued)
| Time | Treatment | Average | Standard error | Label | Cultivars | Gene |
| --- | --- | --- | --- | --- | --- | --- |
| 1d | CK | 1.00 | 0.15 | b | Yunjiao No.1 | NPR1 |
| 1d | TR4 | 0.44 | 0.16 | b | Yunjiao No.1 | NPR1 |
| 1d | TR4+R1 | 3.62 | 0.37 | a | Yunjiao No.1 | NPR1 |
| 1d | TR4+R2 | 0.55 | 0.17 | b | Yunjiao No.1 | NPR1 |
| 7d | CK | 1.00 | 0.07 | b | Yunjiao No.1 | NPR1 |
| 7d | TR4 | 0.62 | 0.09 | b | Yunjiao No.1 | NPR1 |
| 7d | TR4+R1 | 0.66 | 0.16 | b | Yunjiao No.1 | NPR1 |
| 7d | TR4+R2 | 1.22 | 0.17 | a | Yunjiao No.1 | NPR1 |
| 14d | CK | 1.00 | 0.20 | ab | Yunjiao No.1 | NPR1 |
| 14d | TR4 | 0.37 | 0.09 | b | Yunjiao No.1 | NPR1 |
| 14d | TR4+R1 | 1.28 | 0.24 | a | Yunjiao No.1 | NPR1 |
| 14d | TR4+R2 | 1.21 | 0.45 | ab | Yunjiao No.1 | NPR1 |
| 62d | CK | 1.00 | 0.34 | a | Yunjiao No.1 | NPR1 |
| 62d | TR4 | 0.45 | 0.14 | b | Yunjiao No.1 | NPR1 |
| 62d | TR4+R1 | 1.11 | 0.39 | a | Yunjiao No.1 | NPR1 |
| 62d | TR4+R2 | 1.34 | 0.54 | a | Yunjiao No.1 | NPR1 |

## Slide 17
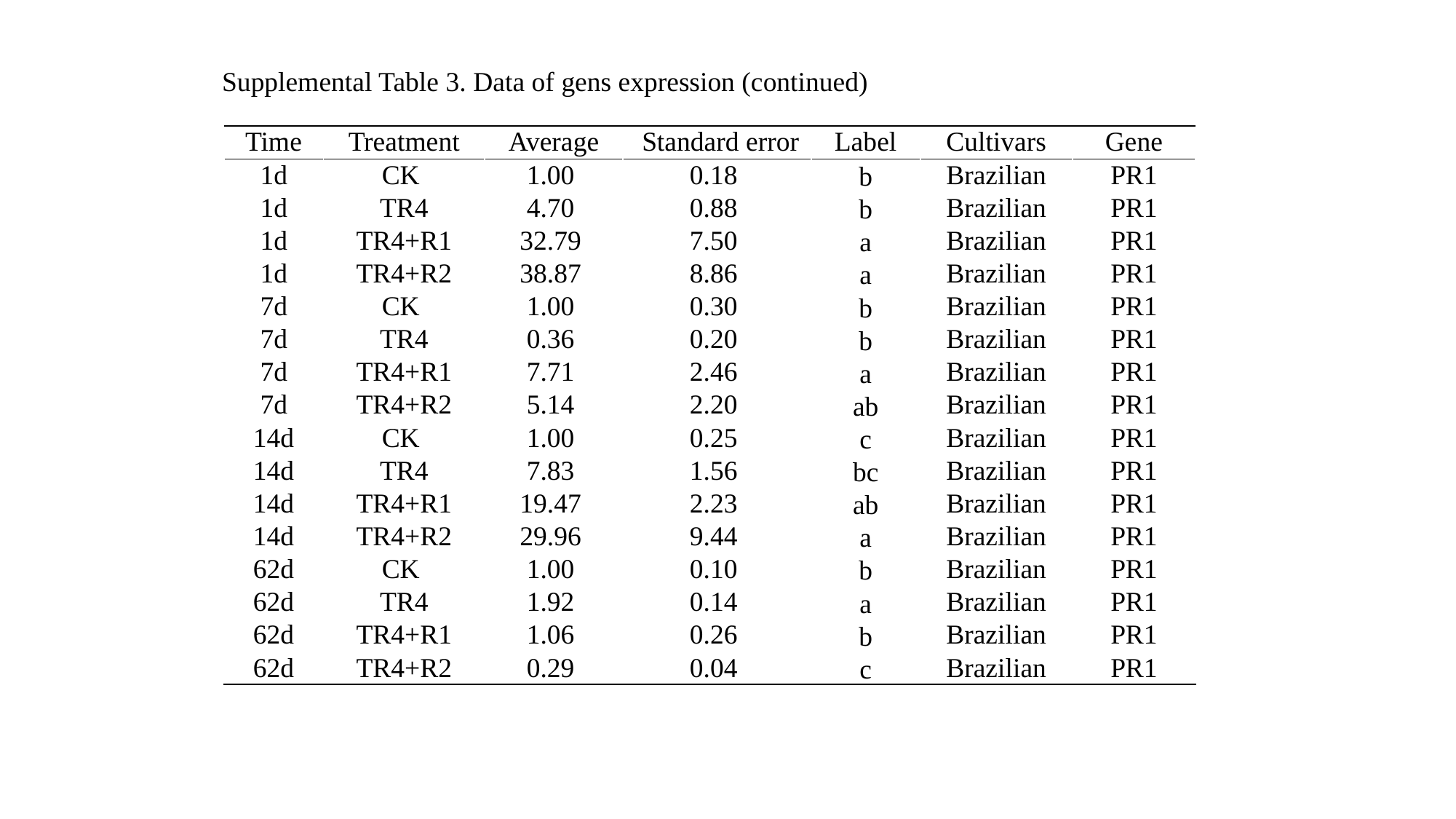

# Supplemental Table 3. Data of gens expression (continued)
| Time | Treatment | Average | Standard error | Label | Cultivars | Gene |
| --- | --- | --- | --- | --- | --- | --- |
| 1d | CK | 1.00 | 0.18 | b | Brazilian | PR1 |
| 1d | TR4 | 4.70 | 0.88 | b | Brazilian | PR1 |
| 1d | TR4+R1 | 32.79 | 7.50 | a | Brazilian | PR1 |
| 1d | TR4+R2 | 38.87 | 8.86 | a | Brazilian | PR1 |
| 7d | CK | 1.00 | 0.30 | b | Brazilian | PR1 |
| 7d | TR4 | 0.36 | 0.20 | b | Brazilian | PR1 |
| 7d | TR4+R1 | 7.71 | 2.46 | a | Brazilian | PR1 |
| 7d | TR4+R2 | 5.14 | 2.20 | ab | Brazilian | PR1 |
| 14d | CK | 1.00 | 0.25 | c | Brazilian | PR1 |
| 14d | TR4 | 7.83 | 1.56 | bc | Brazilian | PR1 |
| 14d | TR4+R1 | 19.47 | 2.23 | ab | Brazilian | PR1 |
| 14d | TR4+R2 | 29.96 | 9.44 | a | Brazilian | PR1 |
| 62d | CK | 1.00 | 0.10 | b | Brazilian | PR1 |
| 62d | TR4 | 1.92 | 0.14 | a | Brazilian | PR1 |
| 62d | TR4+R1 | 1.06 | 0.26 | b | Brazilian | PR1 |
| 62d | TR4+R2 | 0.29 | 0.04 | c | Brazilian | PR1 |

## Slide 18
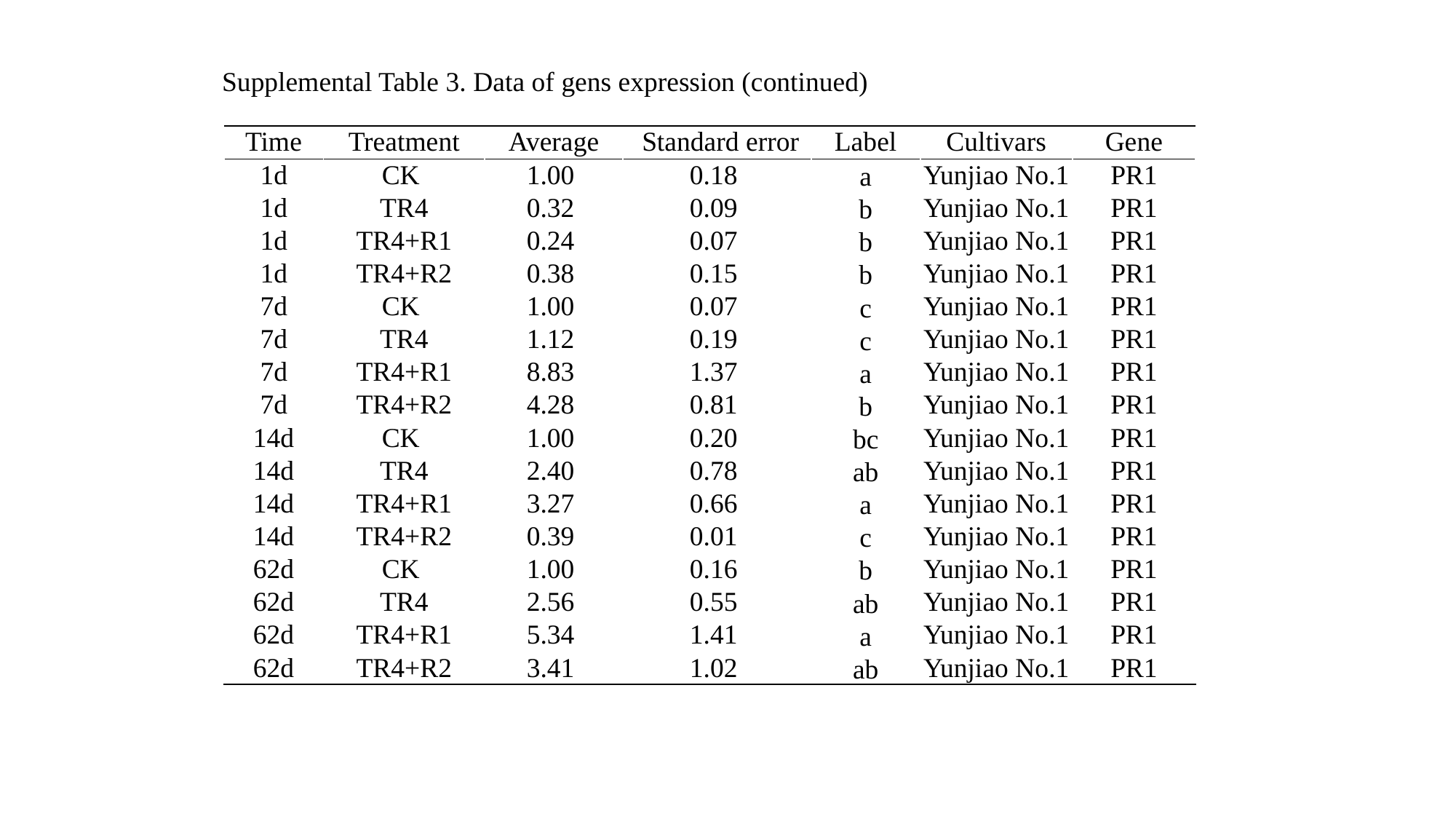

# Supplemental Table 3. Data of gens expression (continued)
| Time | Treatment | Average | Standard error | Label | Cultivars | Gene |
| --- | --- | --- | --- | --- | --- | --- |
| 1d | CK | 1.00 | 0.18 | a | Yunjiao No.1 | PR1 |
| 1d | TR4 | 0.32 | 0.09 | b | Yunjiao No.1 | PR1 |
| 1d | TR4+R1 | 0.24 | 0.07 | b | Yunjiao No.1 | PR1 |
| 1d | TR4+R2 | 0.38 | 0.15 | b | Yunjiao No.1 | PR1 |
| 7d | CK | 1.00 | 0.07 | c | Yunjiao No.1 | PR1 |
| 7d | TR4 | 1.12 | 0.19 | c | Yunjiao No.1 | PR1 |
| 7d | TR4+R1 | 8.83 | 1.37 | a | Yunjiao No.1 | PR1 |
| 7d | TR4+R2 | 4.28 | 0.81 | b | Yunjiao No.1 | PR1 |
| 14d | CK | 1.00 | 0.20 | bc | Yunjiao No.1 | PR1 |
| 14d | TR4 | 2.40 | 0.78 | ab | Yunjiao No.1 | PR1 |
| 14d | TR4+R1 | 3.27 | 0.66 | a | Yunjiao No.1 | PR1 |
| 14d | TR4+R2 | 0.39 | 0.01 | c | Yunjiao No.1 | PR1 |
| 62d | CK | 1.00 | 0.16 | b | Yunjiao No.1 | PR1 |
| 62d | TR4 | 2.56 | 0.55 | ab | Yunjiao No.1 | PR1 |
| 62d | TR4+R1 | 5.34 | 1.41 | a | Yunjiao No.1 | PR1 |
| 62d | TR4+R2 | 3.41 | 1.02 | ab | Yunjiao No.1 | PR1 |

## Slide 19
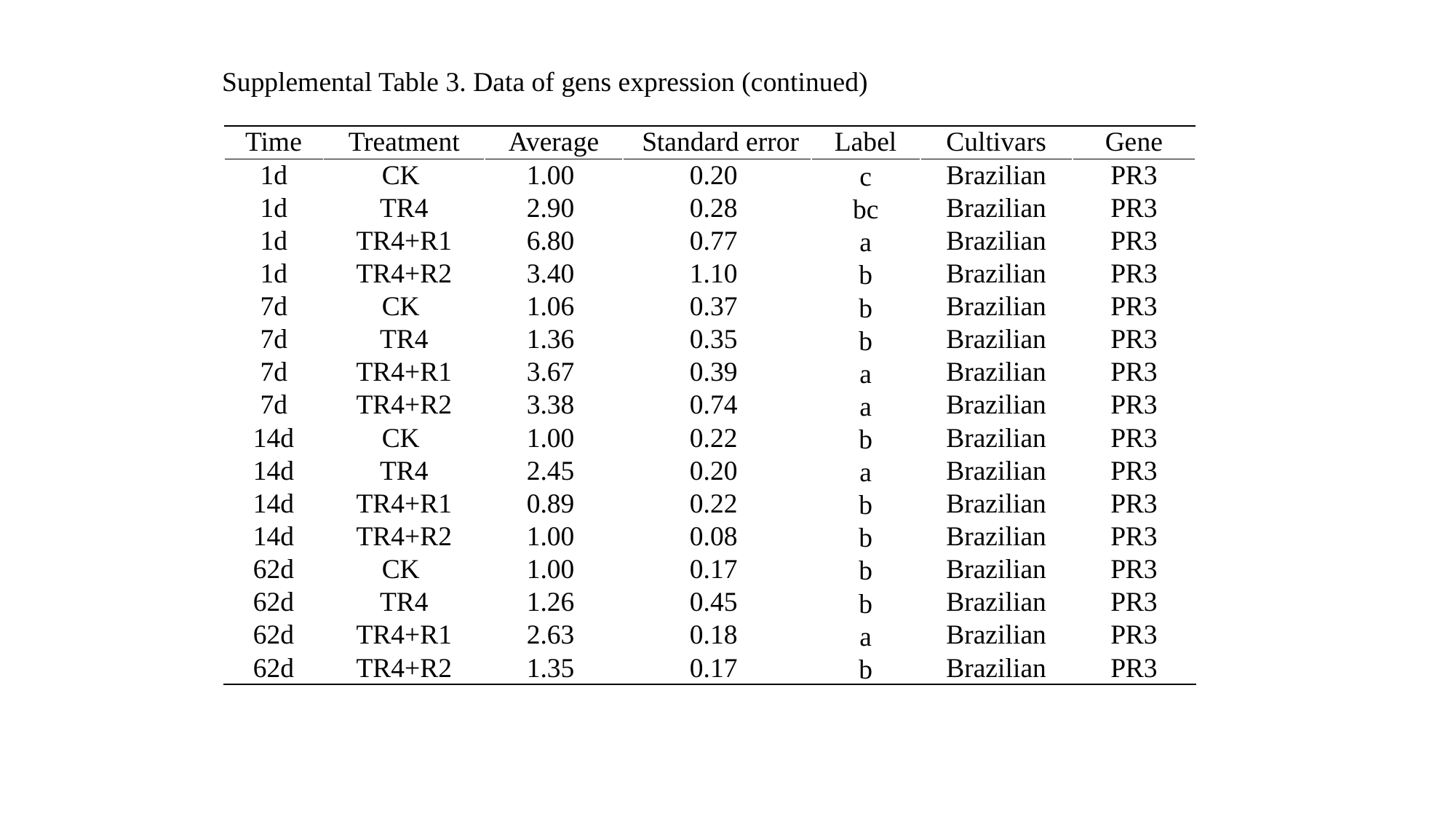

# Supplemental Table 3. Data of gens expression (continued)
| Time | Treatment | Average | Standard error | Label | Cultivars | Gene |
| --- | --- | --- | --- | --- | --- | --- |
| 1d | CK | 1.00 | 0.20 | c | Brazilian | PR3 |
| 1d | TR4 | 2.90 | 0.28 | bc | Brazilian | PR3 |
| 1d | TR4+R1 | 6.80 | 0.77 | a | Brazilian | PR3 |
| 1d | TR4+R2 | 3.40 | 1.10 | b | Brazilian | PR3 |
| 7d | CK | 1.06 | 0.37 | b | Brazilian | PR3 |
| 7d | TR4 | 1.36 | 0.35 | b | Brazilian | PR3 |
| 7d | TR4+R1 | 3.67 | 0.39 | a | Brazilian | PR3 |
| 7d | TR4+R2 | 3.38 | 0.74 | a | Brazilian | PR3 |
| 14d | CK | 1.00 | 0.22 | b | Brazilian | PR3 |
| 14d | TR4 | 2.45 | 0.20 | a | Brazilian | PR3 |
| 14d | TR4+R1 | 0.89 | 0.22 | b | Brazilian | PR3 |
| 14d | TR4+R2 | 1.00 | 0.08 | b | Brazilian | PR3 |
| 62d | CK | 1.00 | 0.17 | b | Brazilian | PR3 |
| 62d | TR4 | 1.26 | 0.45 | b | Brazilian | PR3 |
| 62d | TR4+R1 | 2.63 | 0.18 | a | Brazilian | PR3 |
| 62d | TR4+R2 | 1.35 | 0.17 | b | Brazilian | PR3 |

## Slide 20
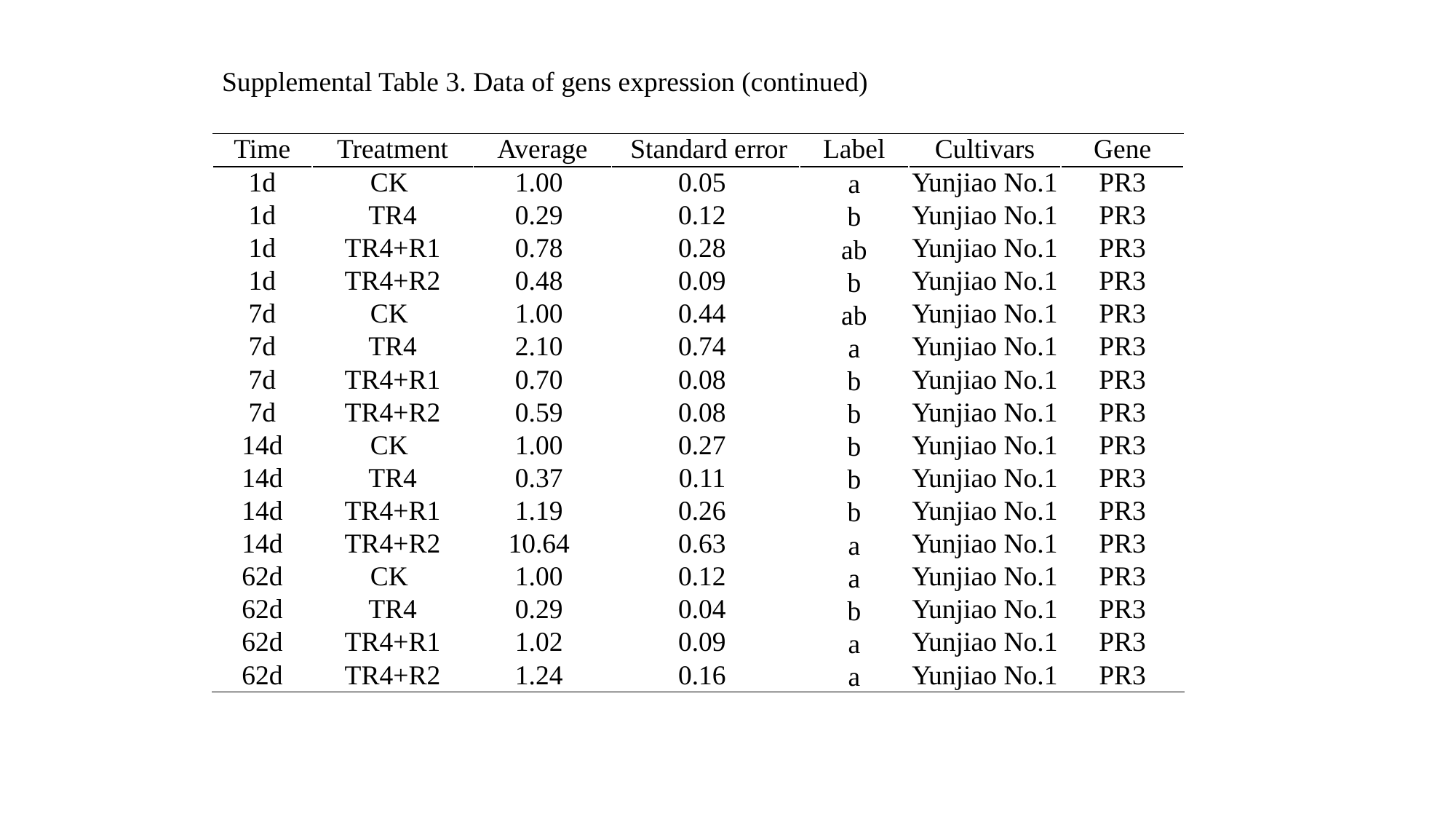

# Supplemental Table 3. Data of gens expression (continued)
| Time | Treatment | Average | Standard error | Label | Cultivars | Gene |
| --- | --- | --- | --- | --- | --- | --- |
| 1d | CK | 1.00 | 0.05 | a | Yunjiao No.1 | PR3 |
| 1d | TR4 | 0.29 | 0.12 | b | Yunjiao No.1 | PR3 |
| 1d | TR4+R1 | 0.78 | 0.28 | ab | Yunjiao No.1 | PR3 |
| 1d | TR4+R2 | 0.48 | 0.09 | b | Yunjiao No.1 | PR3 |
| 7d | CK | 1.00 | 0.44 | ab | Yunjiao No.1 | PR3 |
| 7d | TR4 | 2.10 | 0.74 | a | Yunjiao No.1 | PR3 |
| 7d | TR4+R1 | 0.70 | 0.08 | b | Yunjiao No.1 | PR3 |
| 7d | TR4+R2 | 0.59 | 0.08 | b | Yunjiao No.1 | PR3 |
| 14d | CK | 1.00 | 0.27 | b | Yunjiao No.1 | PR3 |
| 14d | TR4 | 0.37 | 0.11 | b | Yunjiao No.1 | PR3 |
| 14d | TR4+R1 | 1.19 | 0.26 | b | Yunjiao No.1 | PR3 |
| 14d | TR4+R2 | 10.64 | 0.63 | a | Yunjiao No.1 | PR3 |
| 62d | CK | 1.00 | 0.12 | a | Yunjiao No.1 | PR3 |
| 62d | TR4 | 0.29 | 0.04 | b | Yunjiao No.1 | PR3 |
| 62d | TR4+R1 | 1.02 | 0.09 | a | Yunjiao No.1 | PR3 |
| 62d | TR4+R2 | 1.24 | 0.16 | a | Yunjiao No.1 | PR3 |
